# Systematic Comparison of Commercial Uranyl‐Alternative Stains for Negative‐ and Positive‐Staining Transmission Electron Microscopy of Organic Specimens

**DOI:** 10.1002/adhm.202404870

**Published:** 2025-04-29

**Authors:** Vera M. Kissling, Stephanie Eitner, Davide Bottone, Gea Cereghetti, Peter Wick

**Affiliations:** ^1^ Nanomaterials in Health Laboratory Department of Materials Meet Life Swiss Federal Laboratories for Materials Science and Technology (Empa) St. Gallen 9014 Switzerland; ^2^ Centre for Misfolding Diseases Department of Chemistry University of Cambridge Cambridge CB2 1EW UK

**Keywords:** biological samples, negative‐ and positive‐staining transmission electron microscopy, organic nanoparticles, ultrastructure imaging, uranyl‐replacement stains

## Abstract

Negative‐ and positive‐staining transmission electron microscopy (ns/psTEM) is a cornerstone of research and diagnostics, enabling nanometer‐resolution analysis of organic specimens from nanoparticles to cells without requiring costly cryo‐equipment. For nearly 70 years, uranyl salts like uranyl acetate (UA) have been the gold‐standard ns/psTEM‐stains. However, mounting safety concerns due to their high toxicity and radioactivity have led to stricter regulations and expensive licensing requirements. Consequently, there is an urgent global demand for safer, more sustainable stains that deliver uranyl‐comparable, high‐quality ns/psTEM. Here, the commercially available stain‐alternatives UranyLess, UAR, UA‐Zero, PTA, STAIN 77, Nano‐W, NanoVan, and lead citrate are systematically assessed against UA. The stains are evaluated regarding their contrast, resolution, stain‐distribution, and ease‐of‐use in ns/psTEM across a diverse sample set, including polymethylmethacrylate‐nanoplastics, phosphatidylcholine‐liposomes, Influenza‐A viruses, globular ferritin, fibrillar pyruvate kinase amyloids, and human lung‐carcinoma cell‐sections. It is shown that for this variety of samples, a ready‐to‐use uranyl‐alternative is commercially available with comparable or even superior ns/psTEM‐performance to UA using an efficient staining‐protocol. Furthermore, the *GUIDE4U* tool is developed for the fast identification of the appropriate uranyl‐replacements for each sample of interest, saving ns/psTEM‐users time and costs while ensuring excellent staining results for ultrastructural analysis, thereby further catalyzing the use of safer stains.

## Graphical abstract

Negative‐ and positive‐staining TEM is essential for rapid nanometer‐resolution characterization of organic specimens ranging from nanoparticles to cells. Uranyl salts are widely used negative‐/positive‐stains but are radioactive and highly toxic to users and the environment. Safer, commercial alternatives are systematically compared, and effective uranyl‐replacements are identified for a variety of specimens.

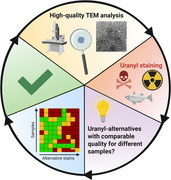

## 1 Introduction

First introduced in the 1950s, negative‐ and positive‐staining transmission electron microscopy (ns/psTEM) is today an essential cornerstone of research and diagnostics for the characterization of organic specimens with low intrinsic scattering contrast.^[^ ^1^, ^2^, ^3^, ^4^, ^5^, ^6^ ^]^ Ns/psTEM has become the gold‐standard technique for rapid, cost‐effective nanometer‐resolution screening and structure analysis at room temperature, as it provides immediate contrast and imaging results without the need for specialized, costly cryo‐equipment or computational resources for extensive post‐acquisition image‐analysis.^[^ ^7^, ^8^, ^9^, ^10^, ^11^ ^]^ For these advantages, ns/psTEM is the method of choice across scientific fields for ultrastructure imaging of biological specimens like cell sections,^[^ ^12^, ^13^, ^14^, ^15^, ^16^, ^17^, ^18^, ^19^ ^]^ bacteria^[^ ^20^ ^]^ or viruses,^[^ ^3^, ^5^, ^20^, ^21^ ^]^ macromolecules like nucleic acids^[^ ^7^, ^22^ ^]^ or amyloid fibrils,^[^ ^18^, ^23^, ^24^, ^25^, ^26^, ^27^ ^]^ soft‐matter nanoparticles like organic synthetic polymer nanoplastics^[^ ^28^ ^]^ or liposomes,^[^ ^6^, ^29^ ^]^ as well as single proteins,^[^ ^9^, ^22^, ^30^ ^]^ despite the recent popularity and rapid technical advancements of atomic‐resolution cryo‐EM.^[^ ^10^, ^31^ ^]^

One of the earliest applications of ns/psTEM was the visualization of viruses, which provided proof of their existence previously only suspected.^[^ ^2^, ^20^ ^]^ Since then, ns/psTEM crucially contributed to the discovery and medical diagnosis of several clinically relevant viruses responsible, among others, for influenza, herpes, smallpox and hepatitis, thereby uniquely allowing the detection of concurring infections by several viruses.^[^ ^3^, ^5^, ^20^ ^]^ Most recently, ns/psTEM was used during the SARS‐CoV‐2 pandemic for its fast identification.^[^ ^3^, ^32^, ^33^, ^34^, ^35^ ^]^ Beyond virology, ns/psTEM is applied in several medical research and diagnostic areas, e.g. to identify genetic disorders like primary cilia dyskinesia^[^ ^4^ ^]^ or to distinguish malignant mesothelioma from lung adenocarcinoma.^[^ ^36^ ^]^ Academic research also continues to rely on ns/psTEM for nanometer‐resolution characterization of soft‐matter, especially particle dispersions of limited concentration or volume, and conformationally flexible^[^ ^10^ ^]^ or oligomerizing samples like DNA‐repair protein complexes^[^ ^22^, ^30^ ^]^ and amyloid fibrils as formed by pyruvate kinase^[^ ^24^, ^25^ ^]^ or α‐synuclein.^[^ ^18^, ^26^, ^27^ ^]^ The rapid, high‐contrast imaging capability of ns/psTEM is further leveraged for screening also prior to atomic‐resolution cryo‐EM in both fundamental and applied research, allowing for example to assess sample purity, heterogeneity, and particle morphology at different conditions, or to visualize cellular changes upon disease, drug treatment or exposure to novel materials.^[^ ^14^, ^16^, ^17^, ^18^, ^19^, ^21^, ^25^, ^31^, ^37^, ^38^, ^39^, ^40^, ^41^, ^42^, ^43^, ^44^ ^]^

For room temperature EM analysis of soft‐matter samples, including cellulose nanocrystals^[^ ^39^ ^]^ in material science or specimens from all kingdoms of life in biology and medicine,^[^ ^3^, ^14^, ^15^, ^16^, ^17^, ^43^, ^44^, ^45^, ^46^, ^47^ ^]^ staining is required due to the intrinsically low scattering contrast of organic materials.^[^ ^9^, ^48^, ^49^ ^]^ Negative‐ or positive‐staining is also needed for the combinational EM‐imaging of newly developed materials, even metal‐based ones, e.g. in 2D/3D human model systems.^[^ ^12^, ^13^, ^19^, ^50^, ^51^, ^52^, ^53^, ^54^, ^55^, ^56^ ^]^ That is because organic materials consist predominantly of light elements such as oxygen, hydrogen, carbon, or nitrogen with low atomic numbers.^[^ ^9^ ^]^ In TEM, they thus scatter the beam electrons in the electron microscope similarly lowly as the surrounding materials (e.g., the grid support film/resin), resulting in very low scattering contrast against the background. In ns/psTEM, the scattering contrast necessary for immediate ultrastructural analysis of organic specimens is achieved via the application of a stain layer, historically consisting of strongly scattering heavy metals like uranium or lead with a high atomic number, onto the organic samples distributed on a TEM grid, or embedded in resin and cut into ultrathin sections on a TEM grid.^[^ ^1^, ^2^, ^5^, ^57^, ^58^, ^59^, ^60^, ^61^ ^]^

**Figure 1:**
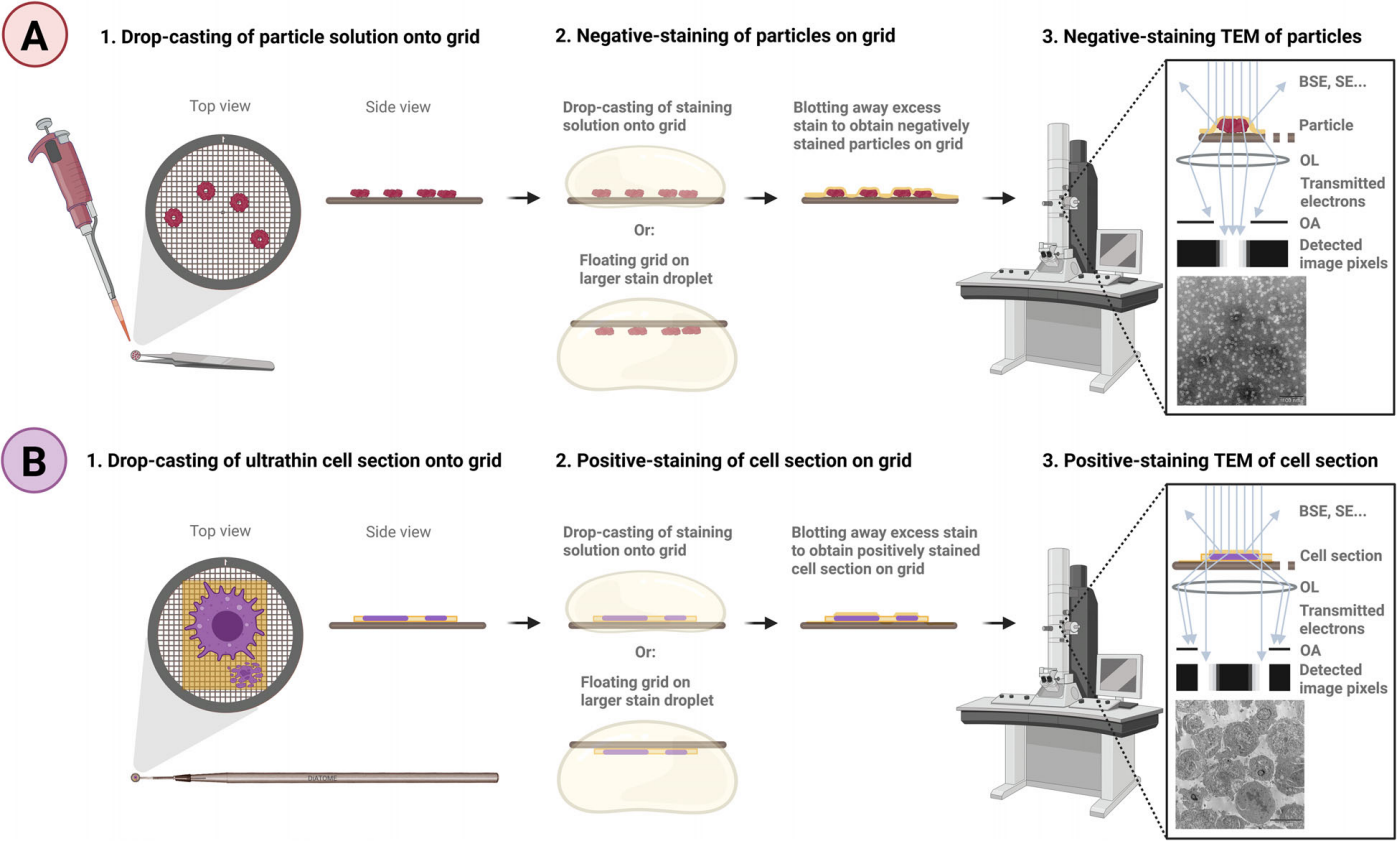
Simplified schematic nsTEM workflow for organic particles in solution/dispersion with ferritin as example (**A**), and psTEM of ultrathin cell sections (here A549 in epoxy) (**B**). Particle drop‐casting via pipette onto TEM grid (Carbon/Formvar), incubating and blotting (**A1**). Cell section drop‐casting on Formvar support grid via Perfect Loop (DiATOME) and air‐drying (**B1**). **A,B,2‐3**: Stain application, blotting, air‐drying and TEM imaging. Imaging is performed with an objective aperture (OA) that removes the transmitted electrons scattered at higher angles by the stain, thereby enhancing the scattering contrast. BSE: Back‐scattered electrons, SE: Secondary electrons, OL: Objective lens. The OA and OL shown in **B3** vary in size solely for better visualization.

In nsTEM, the stain forms a layer around the particles on the grid, leaving them electron‐lucent against a dense background that strongly scatters the incoming beam electrons, creating a negative‐stain contrast in the resulting TEM image (light particles on a dark background) (**Figure** **1**A). Beam electrons that are strongly scattered at large angles by the high z‐atoms of the stains are subsequently removed by the objective aperture, further enhancing the scattering contrast between dark stain and light particles. Yet some samples attract the stain due to electrostatic or hydrophobic interactions, resulting in positive‐staining (dark particles on a lighter background).^[^ ^10^ ^]^ While for instance for some amyloid samples positive‐staining obscures their ultrastructural details like protofibrils and crossovers, psTEM reveals the ultrastructure of cells by highlighting organelles, chromatin, and membranes, with cells appearing darker than the surrounding material (e.g., resin) due to the preferential stain adhesion to cellular material, especially at the section surface^[^ ^5^, ^6^, ^62^ ^]^ (Figure 1B). Beyond increasing scattering contrast, negative‐/positive‐staining can also fix the sample and halt biochemical reactions, allowing to inactivate samples and preserve sensitive or transient sample populations.^[^ ^6^, ^7^, ^9^, ^22^, ^60^, ^63^ ^]^ With the appropriate stain, ≈10‐20 Å resolutions are achieved, sufficient for 2D/3D reconstructions of protein complexes.^[^ ^10^, ^31^, ^38^, ^40^, ^41^, ^42^, ^64^ ^]^

One of the most routinely used stains for ns/psTEM is uranyl acetate (UA) that was first reported by Watson in 1958.^[^ ^1^ ^]^ It has become the gold‐standard due to its reliable ability to produce sharp, highly contrasted electron micrographs within seconds to minutes of incubation time thanks to its small grain size (4‐5 Å) and its strong scattering of the incoming electron beam due to its large atomic number.^[^ ^8^, ^9^, ^10^, ^31^, ^58^, ^65^ ^]^ However, UA solutions are of low pH (4‐5) and were observed to lead to artifacts like stain accumulation, precipitation, particle size overestimation, and structural or beam damage of sensitive samples.^[^ ^5^, ^8^, ^10^, ^20^, ^38^, ^64^, ^66^ ^]^ More recently, uranyl formate (UF) has been suggested especially for particle samples for even better resolution and less artifacts from staining and dehydration, which are the main limitations of ns/psTEM compared to cryo‐EM in near‐native conditions.^[^ ^10^, ^38^, ^57^, ^64^, ^66^ ^]^ Yet, all uranyl salts are radioactive and highly toxic to users and the environment.^[^ ^4^, ^8^, ^9^, ^48^, ^65^, ^67^, ^68^, ^69^ ^]^ Stricter international regulations were recently established for the purchase, transport, use, storage, and disposal of uranium‐based materials, rendering ns/psTEM more cost‐intensive, license‐restricted and requiring special laboratory facilities, which increasingly limits ns/psTEM‐access for many laboratories.^[^ ^4^, ^8^, ^9^, ^48^, ^65^ ^]^ Thus, there is a global and urgent need for alternative stains with lower toxicity and no radioactivity.

A number of uranyl‐replacements have been suggested in the past years, including platinum‐blue (Pt_4_N_8_H_6_O_24_C_20_),^[^ ^70^ ^]^ hematoxylin^[^ ^48^ ^]^ and Ponceau,^[^ ^71^ ^]^ which are themselves suspected to be cancerogenic, mutagenic and toxic to reproduction due to their DNA intercalation, and thus have not been included in this study. Other reagents such as hafnium chloride^[^ ^72^ ^]^ or oolong tea extract^[^ ^73^, ^74^ ^]^ were also assessed as ns/psTEM stain alternatives but were found to perform less well and are less used than UA.^[^ ^4^, ^48^ ^]^ In contrast, the group of lanthanides was lately evaluated regarding their ns/psTEM qualities, as they are close to uranium in their atomic number, and found to harbor promising candidates.^[^ ^4^, ^8^, ^9^, ^58^, ^62^, ^65^ ^]^ While the own production of such stain alternatives in the laboratory is feasible, as shown in recent publications,^[^ ^8^, ^9^, ^58^, ^62^, ^65^ ^]^ commercial ready‐to‐use stains offer major advantages. They ensure higher user‐friendliness, ease‐of‐use, and reproducibility due to lower batch‐to‐batch variability, which is especially important for industrial and clinical laboratories,^[^ ^4^ ^]^ e.g. for compliance with Good Laboratory Practice (GLP) guidelines.

However, their broader adoption is still hindered by a lack of established protocols and guidance on stain selection for specific sample types. To facilitate the shift towards safer non‐radioactive ns/psTEM‐stains, a systematic comparison of commercially available, ready‐to‐use uranyl‐alternatives is needed, evaluating the stains across diverse sample types, independently of the electron microscope's hardware and software configurations. Notably, even the highest‐resolution electron microscope cannot compensate for a sample's lack of high quality or purity and the stain's performance.

Here, we systematically compared the eight commercial, ready‐to‐use ns/psTEM‐stain alternatives UranyLess (denoted as UL, aqueous or in EtOH), UAR, UA‐Zero (UAZ), phosphotungstic acid (PTA), STAIN 77, Nano‐W, NanoVan and lead citrate (Lc) (**Table** **1**). They were selected due to their lower toxicity and lack of radioactivity compared to uranyl salts according to the manufacturer's safety sheets, commercial availability to date (Switzerland, May 2024) and ready‐to‐use packaging (dispenser, septum‐sealed vial or bottle). The stains were evaluated using the same particle solutions/dispersions and staining protocol across specimens as well as a standard TEM with constant hardware and software settings (see Methods section) to ensure the high level of comparability required for the best possible comparison.

**Table 1:** List of uranyl‐alternative stains compared here in ns/psTEM against the reference UA. The active ingredients and pH according to manufacturers and literature are given. The stain short names were generated for better readability.

Stain	Short name	Manufacturer	Active staining ingredients with CAS nr.	pH
Uranyl acetate	UA	Fluka Chemika, CH	Aqueous 2% mixture made with uranyl acetate dihydrate (6159‐44‐0)	4.2–4.6^[^ ^38^ ^]^
Lead citrate	Lc	Electron Microscopy Sciences (EMS), USA	Aqueous <3% mixture of lead (II) nitrate (10099‐74‐8), trisodium citrate dihydrate (<4%, 6132‐04‐3), sodium hydroxide (<1%, 1310‐73‐2) in water (>92%, 7732‐18‐5)	≈12 (em‐grade safety sheet)
UranyLess	UL	EMS, USA	Aqueous 3% lanthanide mixture of dysprosium (7429‐91‐6) and gadolinium (7440‐54‐2), solids: 0%	6.8‐7
Uranyl Acetate Replacement stain	UAR	EMS, USA	Aqueous 15% mixture of the lanthanide salts samarium (7440‐19‐9) and gadolinium triacetate (>2.5‐≤10%, 15280‐53‐2), solids: 0%	7^[^ ^65^ ^]^
UA‐Zero	UAZ	Agar Scientific, UK	Mixture of ytterbium (III) chloride hexahydrate (0.1‐2%, 10035‐01‐5) and phosphotungstic acid hydrate (0.1‐2%, 12501‐23‐4) in ethanol (20%, 64‐17‐5), solids: 74.1‐81.6%	4.6–4.8
Phosphotungstic acid	PTA	EMS, USA	Aqueous 2% mixture of tungstophosphoric acid (12501‐23‐4) made from 3% mixture pH 2.5, solids: 0%	7.5
STAIN 77	–	em‐grade, FR	Mixture of tungsten lithium salts (<1%, 13568‐45‐1) in water (>99%, 7732‐18‐5)	7.6–8.2
Nano‐W^TM^	–	Nanoprobes, USA	Mixture of methylamine tungstate (2% w/w, 55979‐60‐7) in water (98% w/w, 7732‐18‐5)	6.8
NanoVan^TM^	–	Nanoprobes, USA	Mixture of methylamine vanadate (2% w/w, N/A) in water (98% w/w, 7732‐18‐5)	8.0

The uranyl‐alternatives were assessed in their ns/psTEM performance on a broad variety and complexity of soft material and biological sample types with intrinsically low scattering contrast and various sizes, shapes, and compositions to cover a wide spectrum of samples of interest that users might have (**Table** **2**, left column). Accordingly, sample types comprising organic synthetic polymers such as polymethylmethacrylate (PMMA) nanoplastics with and without protein corona as example specimens, lipid membranes like in phosphatidylcholine (POPC) liposomes and the phosphatidylethanolamine (PE)‐rich lipid bilayer within influenza‐A viruses, entire biological entities as for example influenza‐A viruses, nucleic acids like the RNA genome of influenza‐A viruses, globular proteins as e.g. the iron‐loaded ring‐shaped ferritin, fibrillar proteins like the amyloid core of pyruvate kinase (PK) or ultrathin‐sectioned cells like the human adenocarcinoma A549 cells in epoxy were selected for this study (Table 2, left and middle column). For each sample type, these representative example specimens were chosen since they possess distinct intrinsic features with which the stains could be challenged in ns/psTEM (Table 2, middle and right column) and benchmarked against the performance of UA in terms of ns/psTEM‐contrast, resolution, stain‐distribution, ease‐of‐use and when applicable, suitability for size measurements in TEM micrographs, which is common practice in ns/psTEM and depending on sufficient image resolution and contrast to measure the outer particle diameter as accurately as possible.^[^ ^21^, ^28^, ^29^, ^30^, ^37^, ^57^, ^75^ ^]^

**Table 2:** List of the different sample types and their corresponding specific example specimens selected due to their characteristic features to assess the stains in nsTEM for particle samples and post‐staining psTEM of cell sections.

Sample type	Example specimen	Description
Organic synthetic polymers	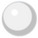 PMMA nanoplastics	**Specimen**: Polymethylmethacrylate (PMMA) nanoparticles **Example for**: Organic, synthetic soft‐matter polymer nanoparticles **Selected as**: One of the most abundant plastics pollutants with low scattering contrast in TEM^[^ ^28^, ^87^, ^88^ ^]^ **TEM features**: ○ Shape/Size: Agglomerates of spherical nanoparticles with well‐detectable diameter of 105 ± 5 nm (mean ± SD, microparticles GmbH) ○ Surface: Smooth with few protrusions ○ Identification of nanoparticle deformation
	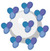 HSA‐corona on PMMA	**Specimen**: PMMA nanoparticles with human serum albumin (HSA)‐protein corona **Example for**: Challenging protein corona‐imaging on nanoparticles with nsTEM^[^ ^89^ ^]^ **Selected as**: HSA is a common component of protein corona on nanoparticles in the blood stream^[^ ^90^ ^]^ **TEM features**: ○ Shape/Size: Agglomerates of spherical PMMA nanoparticles with well‐detectable globular/aggregated HSA ○ Surface: Rough PMMA surface covered with large HSA‐aggregates ○ Identification of corona‐formation
Lipid membranes	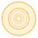 POPC liposomes	**Specimen**: Phosphatidylcholine (POPC) liposomes **Example for**: POPC‐rich mammalian membranes and liposome nanoparticles **Selected as**: Well UA‐stained in nsTEM, mammalian membrane‐like for drug delivery, gene editing etc.^[^ ^8^, ^29^, ^91^ ^]^ **TEM features**: ○ Shape/Size: Non‐homogenized, multi‐membrane liposomes with various shapes/curvatures/thicknesses
	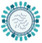 Influenza lipid bilayer on F&C	**Specimen**: Lipid bilayer of H1N1 influenza‐A viruses on Formvar (F) and carbon (C) support grids **Example for**: Phosphatidylethanolamine (PE)‐lipid‐rich membranes inside a biological nanoparticle^[^ ^79^, ^81^ ^]^ **Selected as**: Influenza‐A is one of the most common viruses with a PE‐lipid‐rich membrane^[^ ^79^, ^81^ ^]^ **TEM features**: ○ Shape/Size: Well‐detectable lipid‐bilayer thickness for nsTEM ○ Comparison of membranes with different lipid composition (PE in viruses vs. POPC in mammalian cells)
Biological entities	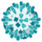 Influenza viruses, especially spike proteins, on F/C	**Specimen**: Inactivated influenza A/Brisbane/59/2007 (H1N1) virus on Formvar (F) or Carbon (C) support grids **Example for**: Biological, nanoparticulate entities and viruses **Selected as**: Influenza‐A is one of the most common viruses and routinely imaged in ns/psTEM^[^ ^21^, ^46^, ^78^, ^92^, ^93^ ^]^ **TEM features**: ○ Shape/Size: Round or elongated, SARS‐CoV‐2‐like, with well‐detectable mean diameter of ≈100 nm^[^ ^93^, ^94^, ^95^ ^]^ ○ Composition: Hemagglutinin (diameter: 3–5 nm, length: 13.5 nm) and neuraminidase (head: 8×8×4 nm, stem: 1.5 nm diameter, 6–10 nm length) spike proteins, PE‐rich bilayer and RNA‐genome^[^ ^77^, ^78^, ^80^, ^81^, ^82^ ^]^ ○ Comparison of different grids (Formvar vs. Carbon support film)
Nucleic acids	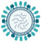 Influenza RNA‐genome on F&C	**Specimen**: RNA‐genome of influenza‐A viruses on Formvar (F) and carbon (C) support grids **Example for**: Challenging nucleic acid‐imaging (even when bound to proteins) with nsTEM^[^ ^7^, ^22^ ^]^ **Selected as**: RNA‐genome is concentrated in viral center and thus more easily found in imaging^[^ ^78^ ^]^ **TEM features**: ○ Shape/Size/Composition: Genome of 8 single‐stranded RNA segments visible as strings within the virus^[^ ^78^ ^]^
Globular proteins	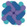 Ferritin (cartoon not true to size for visibility)	**Specimen**: Iron‐loaded horse‐spleen ferritin protein **Example for**: Globular proteins as common nsTEM‐samples **Selected as**: Common TEM reference sample, key for intracellular iron storage, controlled iron release, structure changes linked to metabolic disorders, neurodegeneration and inflammation^[^ ^9^, ^31^, ^75^, ^85^, ^96^, ^97^ ^]^ **TEM features**: ○ Shape/Size: Rings of 24 subunits (length: 5 nm, width: 2.5 nm) with 432‐point symmetry, a mean outer diameter of 11.0 ± 0.7 nm (±SD) and an occasionally visible dense core of up to 4500 Fe^3+^ ions^[^ ^75^, ^83^, ^84^, ^85^ ^]^ ○ Assessment of imaging resolution due to small protein size and stain‐induced preferred particle orientation
Fibrillar proteins	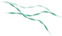 PK amyloid core (cartoon not true to size for visibility)	**Specimen**: Amyloid core peptide of yeast pyruvate kinase (PK) **Example for**: Challenging fibrillar/amyloid protein‐imaging with nsTEM^[^ ^37^ ^]^ **Selected as**: It forms stable, reversible amyloid fibrils essential for cell survival yet structurally resembles irreversible pathological amyloids linked to neurodegeneration, metabolic disorders, and cancer^[^ ^24^, ^25^, ^98^, ^99^ ^]^ **TEM features**: ○ Shape/Size: Up to micrometer‐long amyloid fibrils with protofibrils twisting at regular crossovers^[^ ^24^, ^25^ ^]^ ○ Predisposition to polymorphism, clustering, stain accumulation, and positive‐staining^[^ ^24^, ^25^, ^37^ ^]^
Ultrathin cell sections	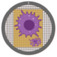 A549 cells (cartoon not true to size for visibility)	**Specimen**: Human alveolar basal epithelial cells from non‐small lung adenocarcinoma (A549) in epoxy resin **Example for**: Ultrathin sections of cells commonly imaged in psTEM on Formvar support grids **Selected as**: Common primary cell model with typical eukaryotic cell ultrastructure^[^ ^50^, ^51^, ^86^ ^]^ **TEM features**: ○ Shape/Size: Several tens of micrometers in diameter^[^ ^86^ ^]^ ○ Composition: POPC‐rich cell membrane, lipids, proteins, nucleic acids, etc. and common organelles^[^ ^86^ ^]^ ○ Comparison of staining of different organelles, e.g. mitochondria vs. Golgi/ER

Notably, in the transmission electron microscope, contrast is generated through the combination of scattering and phase contrast.^[^ ^76^ ^]^ The scattering contrast is the focus of this study, since it is enhanced with the use of stains in ns/psTEM. We define high (scattering) contrast in this work as a large difference in grey‐scale intensity between the particles and the background in an image. That would be the case for example when the ferritin rings are bright, white, and well discernable from the dark grey stain background and the central iron core. While scattering contrast depends heavily on the objective aperture size, the contribution of phase contrast to the image contrast depends on the defocus settings.^[^ ^76^ ^]^ On the other hand, the resolution of the image is influenced by both the objective aperture size and defocus.^[^ ^76^ ^]^ Thus, the objective aperture size and defocus range were kept constant for all samples to be able to best compare the contribution of the ns/psTEM‐stains to the scattering contrast and resolution of the images (see Methods).

The resolution of the images produced with the different stains was compared here based on the visibility and sharpness of specific ultrastructural features of the respective sample (Table 2). For PMMA nanoparticles we assessed the outer particle borders and the HSA proteins on the PMMA surface for HSA‐PMMA, while for the POPC liposomes we evaluated how well and sharp the lipid membranes were visible. For influenza‐A viruses we rated the visibility and sharpness of the spike proteins.^[^ ^77^, ^78^, ^79^, ^80^, ^81^ ^]^ For ferritin rings we assessed how well and sharp their outer borders as well as their subunits were visible.^[^ ^75^, ^83^, ^84^, ^85^ ^]^ For PK amyloid fibrils, the visibility and sharpness of the protofibrils and crossovers was evaluated, while in the lung cells we focused on certain organelle structures such as the ER/Golgi membranes and the mitochondrial cristae.^[^ ^50^, ^51^, ^86^ ^]^ Furthermore, the size measurements performed for certain samples served as an additional measure for sufficient image resolution and contrast to be able to discern the particles well from the background and reproduce the reference sizes as accurately as possible. Additionally, the highest magnification nsTEM images of ferritin were subjected to a frequency domain analysis and compared with reference synthetic data of ferritin rings to further assess the resolution achieved with the different stains. As the structural complexity and irregularity found typically in organic samples, e.g. influenza‐A viruses with varying shapes, diameters and suborganizations (e.g., spike proteins, lipid bilayer, genome), significantly complicate such a frequency domain analysis and the generation of synthetic data for further resolution assessment, we focused on ferritin in this study as an example due to its use as organic TEM‐reference particle and its regular structure.^[^ ^83^ ^]^

Furthermore, we used a simple and safe conventional ns/psTEM staining method with a short staining time to provide users of various experience levels an easy and fast protocol as a starting point. We optimized this method to allow straightforward, effective and efficient work, eliminating the need for high‐end or additional equipment, such as a glow discharger.

While these sample types and example specimens were selected to represent a broad spectrum of known user samples commonly studied with ns/psTEM, and to our knowledge this is the first systematic comparison including such a wide range of sample types and commercial stains in one study, also other sample types not assessed here could be investigated with the uranyl‐alternatives. We further used two different grids (carbon vs. Formvar support film) to identify any differences in support film‐stain interactions and stain‐distributions depending on the support film material. Additionally, we assessed one stain, UL, available in water or ethanol, regarding solvent‐based differences in stain penetration of ultrathin A549 epoxy sections.

Based on the results of this study, we developed the multi‐dimensional platform *GUIDE4U* (see Table 3) for users of varying ns/psTEM‐experience to easily select the suitable uranyl‐replacement stains for their samples with a straightforward staining protocol provided as a basis for further fine‐tuning. This significantly reduces the stain screening and optimization time as well as costs for ns/psTEM‐users, catalyzing the targeted acquisition and use of the appropriate, safer stains without special licenses required.^[^ ^9^ ^]^

Our findings encourage a more wide‐spread use of uranyl‐replacement stains in various research disciplines for a broad range of specimens as well as for diagnostic procedures in clinical settings thanks to their commercial availability. By supporting a minimized application of uranyl salts in the ns/psTEM‐community, this study significantly advances the general trend to greener, less toxic chemistry also in the field of electron microscopy.

## 2 Results and Discussion

### 2.1 Negative‐Staining of Organic Polymethylmethacrylate (PMMA)‐Nanoplastics

**Figure 2:**
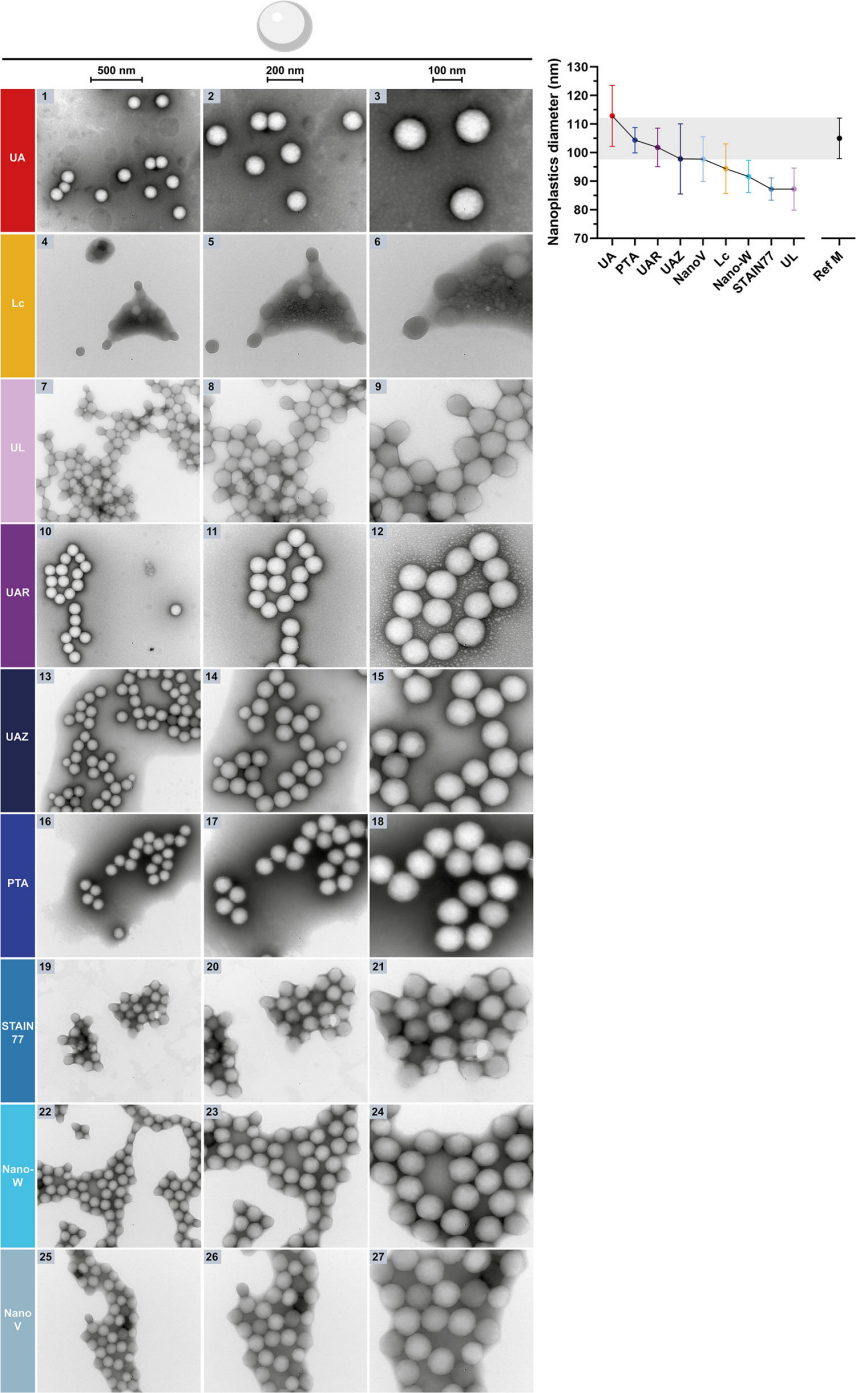
Left: NsTEM of PMMA nanoplastics on carbon support grids with uranyl‐alternative stains benchmarked against UA. The three columns show the sample at increasing magnifications (see scale bars: 500, 200, 100 nm). The nanoplastic particles and their uneven surface are visible. Right: Plot of the mean PMMA particle diameters ± SD from nsTEM images as in Figure 2 (left). The manufacturer reference is shown as grey area.

First, the uranyl‐alternatives were assessed in their nsTEM performance compared to UA (**Figure** **2**, Left, Images 1–3 and zoom‐inset) on PMMA nanoplastics spread on a carbon support grid as an example for organic, synthetic soft‐matter nanoparticles with intrinsically low scattering contrast.^[^ ^28^ ^]^ In nsTEM of PMMA, Lc provided scattering contrast, but highly uneven stain‐distribution, low resolution (i.e., particle borders are not well visible), beam damage as well as particle shrinking artifacts (Figure 2, 4–6). Conversely, UL resulted in lighter staining with sufficient contrast and resolution for a TEM characterization (i.e., nanoplastics appear darker than the grid background and the particle borders are well visible), yet also induced particle shrinking to squeezed and more rectangular PMMA shapes (Figure 2, 7–9). Interestingly, UAR, UAZ, and PTA showed similar stain contrast and PMMA morphology, visualizing the nanoparticle surface and its unevenness with high resolution (Figure 2, 10–18). While UAZ and especially PTA achieved darker staining, similar to UA, they also distributed more unevenly on the carbon grid than UAR. PTA further produced a lightly visible stain ring spanning into the particle spheres, hindering higher resolution imaging of the particle surface compared to UAR and UAZ. STAIN 77 and Nano‐W resulted in similar staining darkness and contrast, yet STAIN 77 coated the particles more unevenly and achieved lower resolution (Figure 2, 19–27). Also, Nano‐W allowed slightly higher resolution imaging of the uneven nanoparticle surfaces and produced higher contrast than NanoV that has lower contrast by design for immunogold TEM (Nanoprobes). STAIN 77, Nano‐W, and NanoV concentrated at the PMMA agglomerates, leading to more uneven stain‐distribution on the grid.

Thus, UAR resembled UA contrast‐, resolution‐ and stain‐distribution‐wise the most, allowing a similar imaging of the particle surface, followed by UAZ despite its uneven stain‐distribution on the grid.

In addition, the diameters of individual PMMA particles were measured at their longest axis in the TEM micrographs in Figure 2 (left) for a particle size analysis as commonly performed in nsTEM and material science.^[^ ^21^, ^28^, ^29^, ^30^, ^37^, ^57^, ^75^ ^]^ In UA‐stained images, a mean PMMA diameter of 112.9 ± 10.7 nm (±SD) was measured, 104.4 ± 4.4 nm in PTA, 101.8 ± 6.7 nm in UAR, 97.8 ± 11.3 nm in UAZ, 97.7 ± 7.8 nm in NanoV, 94.4 ± 8.7 nm in Lc, 91.6 ± 5.6 nm in Nano‐W, 87.2 ± 3.9 nm in STAIN 77 and 87.2 ± 7.4 nm in UL. Compared to the mean particle diameter of 105 ± 5 nm determined by the manufacturer using different methods including TEM and indicated as grey area in Figure 2 (right), the mean particle diameters measured with PTA and UAR staining were within that range including at least one SD error bar, while UA resulted in measurements with higher inaccuracy (larger SD) and a mean bordering to size overestimation ‒ one of the artifacts observed with UA due to stain‐accumulation or flattening of particles.^[^ ^5^, ^8^, ^10^, ^20^, ^38^, ^64^, ^66^ ^]^ UAZ and NanoV also had one SD error bar within the reference range, but their mean size tended towards an underestimation like most of the uranyl‐alternatives assessed here, and UAZ measurements suffered from a notably higher inaccuracy (larger SD).

**Figure 3:**
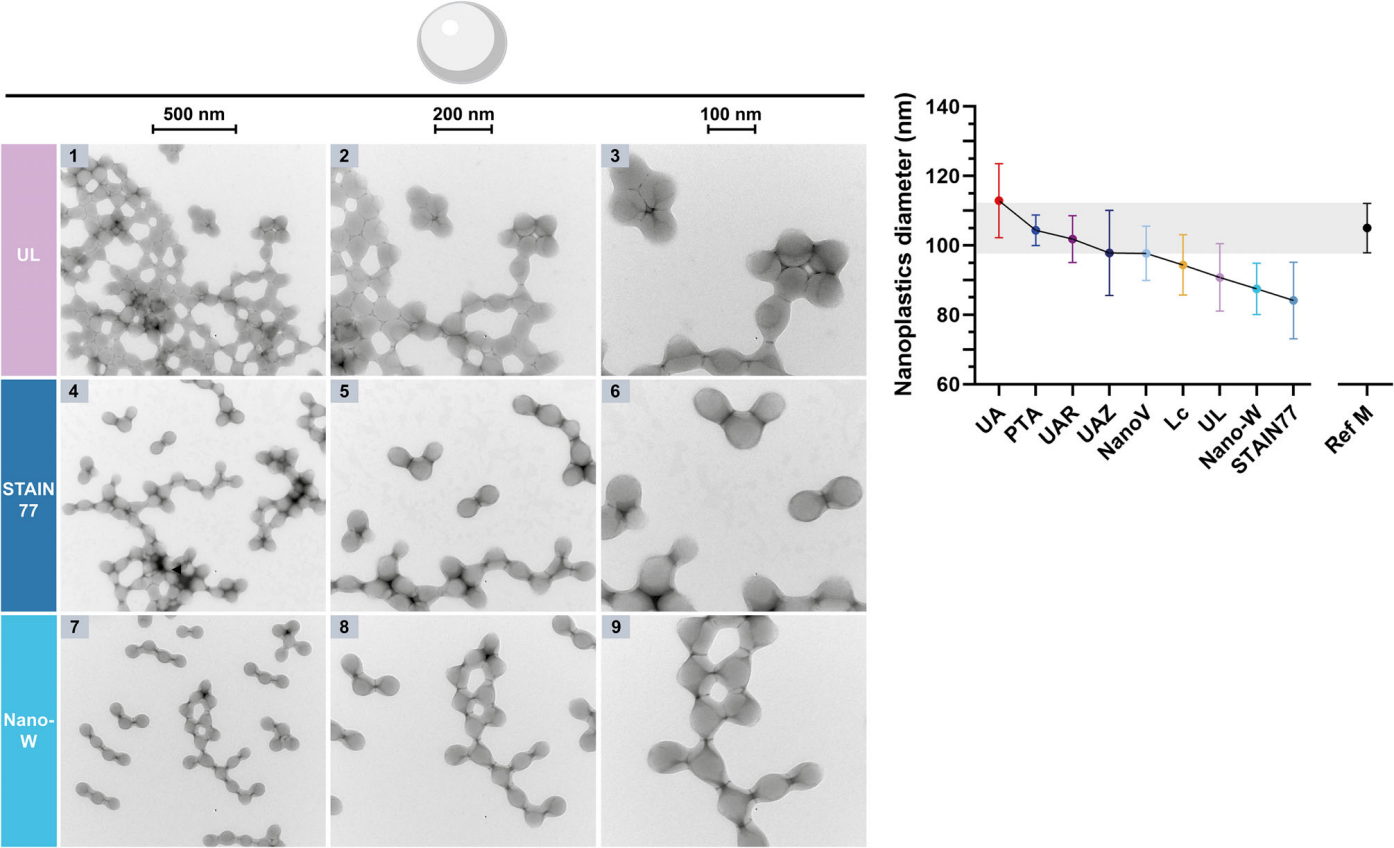
Left: PsTEM of PMMA nanoplastics on carbon support grids abundantly observed with uranyl‐alternatives UL, STAIN 77, and Nano‐W. The three columns show the sample at increasing magnifications with the shrinking and swelling artifacts in nanoparticle size visible compared to Figure 2 (see scale bars: 500, 200, 100 nm). Right: Plot of the mean PMMA particle diameters ± SD from nsTEM micrographs as in Figure 2 and psTEM images as in Figure 3 (left) for UL, STAIN 77, and Nano‐W. The manufacturer reference is shown as grey area.

For some stains, positive‐staining of the nanoplastics was also abundantly visible on the grids, where the particles appeared much darker stained than the background (**Figure** **3**, left). Comparing the diameters from these positively stained PMMA particles to the negatively stained nanoplastics revealed shrinking and swelling artifacts (Figure 3, right). Smaller mean particle diameters were found in STAIN 77 (84.2 ± 11.0 nm) and Nano‐W (87.5 ± 7.4 nm) with positive‐staining in Figure 3 compared to negatively stained PMMA in Figure 2, and larger particle diameters were found for UL (90.8 ± 9.7 nm) with positive‐staining. However, also in the images considered as negatively stained in Figure 2, UL, STAIN77, Nano‐W, and NanoV stained the particles and background so unevenly that the staining resembled positive‐staining.

In summary, PTA resulted in the highest accuracy for diameter measurements, yet also in uneven stain‐distribution and stain accumulations as rings on the particles. Notably, inhomogeneous stain‐distribution has been reported previously for PTA but also UA, originating from stain properties, interactions with the support film, and the staining process itself.^[^ ^8^, ^10^ ^]^ Conversely, UL, STAIN 77, and Nano‐W showed positive‐staining and resulted in more inaccurate diameter measurements even in negative‐staining due to shrinking artifacts (Figures 2 and 3). UAZ and NanoV were barely in the reference size range and additionally showed uneven stain‐distribution. The range of measured mean sizes was rather large with the difference between the smallest (STAIN 77, ≈87 nm) and the largest mean size (UA, ≈113 nm) being almost a quarter (≈26 nm) of the particle size reported by the manufacturer (105 nm). Such large deformation of PMMA may originate from its water content changing during imaging due to heating in the beam and insufficient protective coating of the nanoplastics by the stains, or tension forces during air‐drying of the grids as well as stain components affecting the PMMA material.^[^ ^8^, ^10^, ^64^ ^]^ Possibly, the 15% lanthanide content of UAR provided stronger protection against PMMA deformation, especially with its samarium component, since gadolinium is also present in UL yet to a lower extent. Also phosphotungstic acid seems to prevent PMMA deformation as seen in PTA, but not as efficiently when combined with e.g. ytterbium or dissolved in ethanol as in UAZ.^[^ ^58^ ^]^ Other forms of tungsten as in STAIN 77 or Nano‐W, or a different pH of the stain could not prevent PMMA deformation.

Taking all criteria together, UAR provided the most comparable nsTEM contrast, resolution, and stain‐distribution for PMMA to UA, while allowing even more accurate size measurements than UA (mean within reference range, smaller SD), rendering UAR an excellent uranyl‐replacement stain for polymer nanoparticles such as PMMA (see Table 3).

### 2.2 Negative‐Staining of Human Serum Albumin (HSA)‐Protein Corona on PMMA‐Nanoplastics

**Figure 4:**
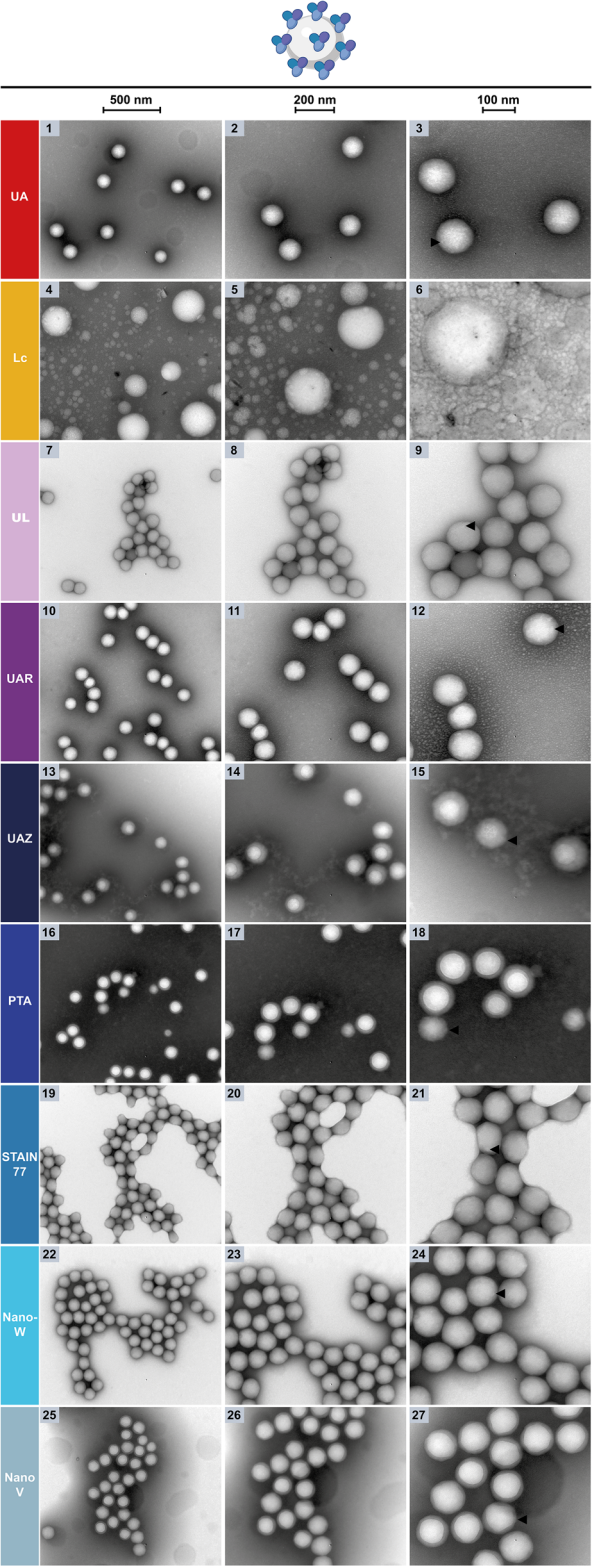
NsTEM of PMMA nanoplastics with HSA‐protein corona on carbon support grids. The three columns show the sample at increasing magnifications (see scale bars: 500, 200, 100 nm) with HSA‐protein aggregation visible on the nanoparticles' surface (black arrows).

Next, we investigated the nsTEM‐performance of the uranyl‐alternatives benchmarked against UA (**Figure** **4**, 1–3) when a protein corona of human serum albumin (HSA) is present on the surface of PMMA nanoplastic particles, which is difficult to simultaneously visualize as well as the nanoparticles.^[^ ^89^ ^]^

In nsTEM of HSA‐PMMA, Lc provided contrast but led to swelling of the nanoparticles as well as other staining artifacts in the form of white droplets, rendering the detection of clear HSA aggregates on PMMA impossible (Figure 4, 4–6). Conversely, UL resulted in lighter staining with sufficient contrast and resolution for a TEM characterization as for PMMA nanoparticles alone (Figure 2), but also induced again slight particle shrinking to more squeezed, rectangular shapes rather than round spheres (Figure 4, 7–9). Yet, lightly stained, white aggregates and uneven PMMA particle surfaces were visible in the HSA‐PMMA sample with UL, hinting to HSA aggregates on the nanoplastics. Interestingly, UAR, UAZ, and PTA produced again similar stain contrast, which was even darker than for PMMA alone (Figures 2 and 4, 10–18). Furthermore, a stain ring extending into the particle spheres was already lightly visible in PMMA, but now increased for HSA‐PMMA in UAZ and PTA. While few lightly white spots were observed on PMMA particles alone in UAR, these white spots increased in abundance, size, protrusion from the particles and visibility, showing HSA aggregates on the PMMA surfaces (Figures 2 and 4, 10–12). Clear HSA presence and binding to PMMA nanoplastics were also detected with UAZ in the form of white aggregated particles with typical protein morphology appearing in the background and on the PMMA surfaces (Figures 2 and 4, 13–15).

The stain rings on the nanoplastics were most pronounced in PTA and obscured any HSA protein corona at these locations, however, a general unevenness and fuzziness of the PMMA surfaces were observed, as well as occasionally clearly protein‐covered spheres (Figures 2 and 4, 16–18). STAIN 77 and Nano‐W produced again a lighter stain compared to the other reagents, while NanoV resulted in a darker, yet more unevenly distributed staining with HSA in the sample, in contrast to all other stains that covered the grids more evenly for HSA‐PMMA than PMMA alone, likely due to the protein excess and its buffer solution in the sample hydrophilizing the grid (Figures 2 and 4, 19–27). While STAIN 77 stained PMMA alone unevenly and at lower resolution, it resolved the nanoparticle surface and its unevenness well for HSA‐PMMA, including white HSA aggregates on the PMMA spheres (Figures 2 and 4, 19–21). However, STAIN 77 induced PMMA shrinking also in presence of HSA since nanoplastics appeared squeezed together. Nano‐W resolved some white dots on the PMMA nanoplastics without HSA in the sample, but a potential slight increase in their number and protrusion was hard to observe in HSA‐PMMA (Figures 2 and 4, 22–24). Similarly, NanoV showed uneven nanoparticle surfaces in PMMA alone, which remained similar in the HSA‐PMMA sample, and any protein aggregations were difficult to resolve, also due to the dark stain rings covering the particle spheres (Figures 2 and 4, 25–27). Interestingly, also for UA, white dots and uneven surfaces were already visible for PMMA alone, and only a slight increase in abundance and visibility as well as a size decrease of these dots was observable for HSA‐PMMA (Figures 2 and 4, 1–3).

In general, less PMMA deformation was observed with HSA in the sample, probably due to a change in charge and composition of the PMMA surface owing to the protein corona likely altering the interactions of the stain components with the PMMA particles.^[^ ^10^ ^]^ Also for HSA‐coated PMMA particles, the 15% lanthanide content of UAR and especially its samarium component possibly provided stronger protection against PMMA deformation, allowing to resolve the protein corona well. Phosphotungstic acid in PTA and UAZ achieved a similar effect for HSA‐PMMA that was more pronounced than for PMMA alone, but did not interact as favorably as UAR with the HSA‐PMMA surface and excess HSA on the grid, leading to more uneven stain‐distribution. Again, other stain components or forms of tungsten as well as a different pH of the staining solution could not prevent PMMA deformation, uneven stain‐distribution, or inferior staining of the protein corona.

In summary, UAR again proved to be a suitable uranyl‐replacement for nsTEM protein corona visualization on organic nanoparticles, providing even better visibility of HSA aggregation on PMMA nanoplastics than UA (see Table 3). As UAR and other nsTEM‐stains like UAZ and PTA were clearly able to resolve HSA on the PMMA surface as fuzzy white aggregates, such an nsTEM analysis could be useful for the rapid screening of HSA‐PMMA binding conditions, while high‐resolution imaging of the protein corona on nanoparticles is best performed using cryo‐TEM as demonstrated by Sheibani et al.^[^ ^89^ ^]^

### 2.3 Negative‐Staining of Multi‐Membrane Phosphatidylcholine (POPC)‐Liposomes

**Figure 5:**
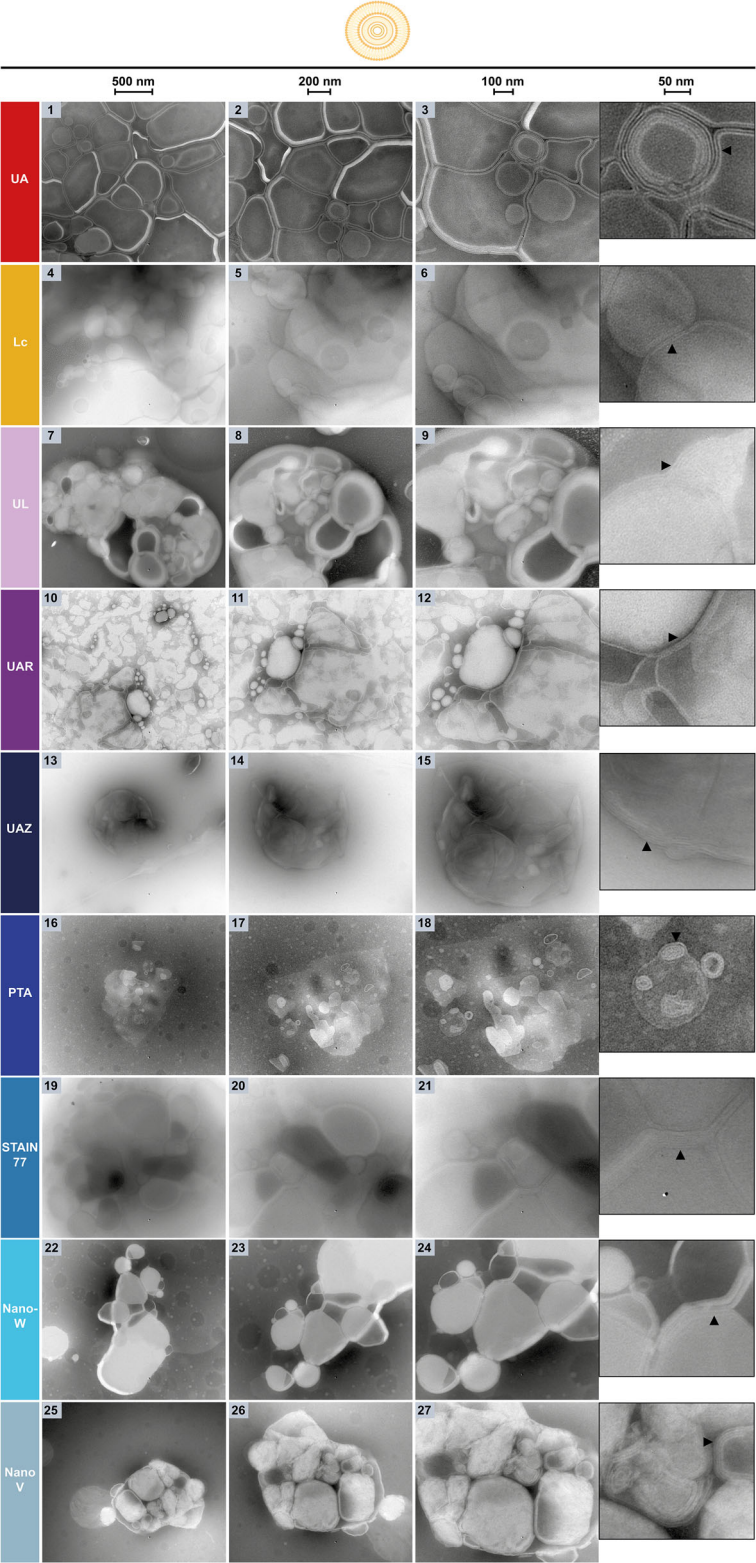
NsTEM of POPC‐liposomes on carbon support grids. The three columns and zoom‐insets show the sample at increasing magnifications (see scale bars: 500, 200, 100, 50 nm) with multilamellar liposomes and their individual lipid membranes visible (black arrows).

After assessing the nsTEM‐performance of the uranyl‐alternatives on soft‐matter polymer nanomaterials such as PMMA, the stains were next evaluated against UA (**Figure** **5**, 1–3 and zoom‐inset) for another type of nanoparticles highly relevant for research and clinics: non‐homogenized phosphatidylcholine (POPC) liposomes on carbon support grids as an example of lipid‐rich and mammalian membrane‐like samples, which are usually very well stained by UA.^[^ ^8^, ^29^ ^]^

In nsTEM of POPC, Lc produced contrast and negative‐staining with even stain‐distribution. Beam damage was interestingly lower than for the previous samples, allowing the visualization of the liposomal membranes of the differently shaped and sized liposomes, yet at low resolution (Figure 5, 4–6 and zoom‐inset). UL, PTA, Nano‐W and NanoV achieved a similar stain darkness in the lower magnification images, while STAIN 77 followed by UAZ and UAR produced a lighter stain (Figure 5, 7–27). STAIN 77, succeeded by UAZ and UL, showed sufficient resolution to visualize the individual membranes of multilamellar POPC liposomes, especially at higher magnification, yet at lower contrast, while Nano‐W showed inferior resolution (Figure 5, 7–9, 13–15, 19–24 and zoom‐insets). In comparison, UAR stained the membranes with higher contrast and similar if not superior resolution as visible in the high magnification zoom‐inset, despite overall lower stain darkness (Figure 5, 10–12 and zoom‐inset). PTA and NanoV achieved even higher contrast and stain darkness, with PTA showing slightly lower resolution and NanoV marginally lower contrast. NanoV reached the highest resolution of the multilamellar membranes of all uranyl‐alternatives as especially visible in the high‐resolution zoom‐inset (Figure 5, 16–18, 22–27 and zoom‐insets). All stains were distributed evenly on the carbon support grid for this sample.

Taken all together, UA‐stained liposomes in terms of contrast, resolution, and stain darkness exceptionally well, but the lanthanide‐based UAR, and transition metal‐based PTA and NanoV stains reached a sufficiently similar nsTEM‐performance for the characterization of liposomal morphology and the visualization of individual membranes of multilamellar POPC liposomes (see Table 3). The slightly lower resolution of PTA than UA could be explained by its larger grain size of 8–9 Å vs. 4–5 Å of UA,^[^ ^10^ ^]^ while NanoV's lower contrast was by design. UAR resulted in lower stain darkness than UA, but nonetheless allowed high contrast‐ and resolution‐imaging of POPC‐based membranes.

### 2.4 Negative‐Staining of Inactivated Influenza‐A Viruses on Formvar Support Grids, including Spike Proteins, Phosphatidylethanolamine (PE)‐Rich Lipid Bilayer and RNA‐Genome

**Figure 6:**
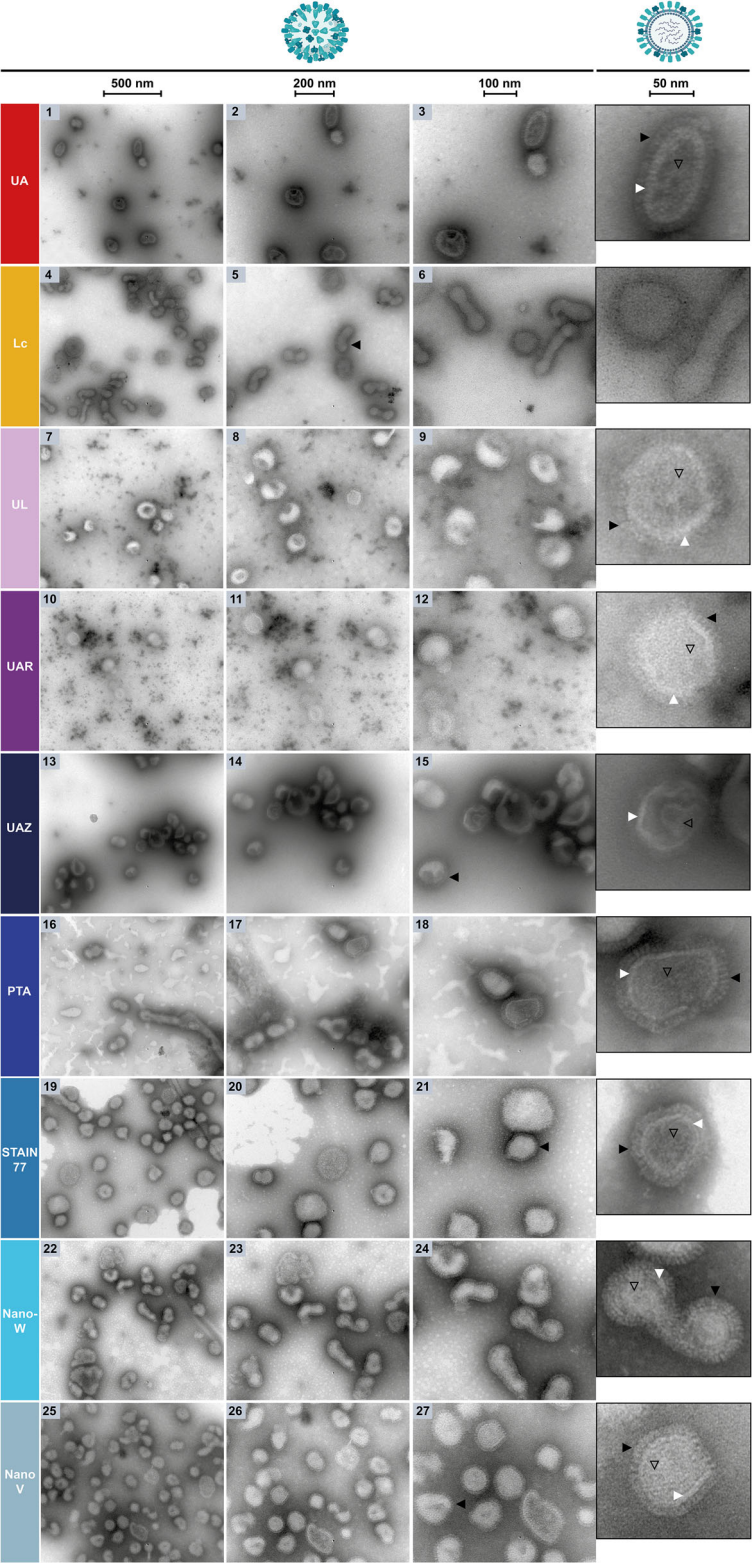
NsTEM of inactivated influenza‐A virus on Formvar support grids. The three columns and zoom‐insets show the sample at increasing magnifications (see scale bars: 500, 200, 100, 50 nm). The viruses and their surface spike proteins are visible (black arrows), as well as their lipid bilayer (white arrows) and internal RNA‐genome (black outlined arrows), as shown schematically in the cartoons.

The nsTEM‐analysis of inactivated influenza‐A viruses on Formvar support grids as an example of nanoparticulate biological entities and viruses revealed that in comparison to UA (**Figure** **6**, 1–3), Lc provided contrast, even stain‐distribution on the grid, and staining of the outer border of the virus particles, yet did not allow the visualization of the hemagglutinin and neuraminidase spike proteins on the viral surface, the PE‐rich lipid bilayer or the RNA‐genome inside the viruses^[^ ^77^, ^78^, ^80^, ^81^, ^82^ ^]^ (Table 2, Figure 6, 4–6). Moreover, beam damage in the form of black dots appeared at higher magnifications (Figure 6, 6). The lanthanide‐based UL and UAR stains both produced lighter negative‐staining contrast and stain precipitates (Figure 6, 7–12). This likely originated from the interaction between gadolinium and phosphate of the PBS buffer of the virus sample, forming insoluble gadolinium phosphate (GdPO_4_) precipitates that could not be removed even with water washes.^[^ ^10^, ^100^ ^]^ Moreover, the resolution of UL was inferior for a suitable TEM characterization of the viral PE‐rich lipid bilayer and the spike proteins were not well visible (Figure 6, 7–9 and zoom‐inset).

A slightly higher resolution was achieved with UAR, resulting in a clearer visualization of the lipid bilayer and the individual spike proteins, but not as well as with other UA‐alternatives (Figure 6, 10–12 and zoom‐inset). With both, UL and UAR, the RNA‐genome inside the viruses was difficult to resolve (Figure 6, 9,12 and zoom‐insets). UAZ provided stronger contrast, potentially owing to its transition‐metal component phosphotungstic acid in addition to its lanthanide content as UL and UAR, yet due to the dark stain accumulation around the viruses, higher resolution imaging of the individual spike proteins was obstructed (Figure 6, 13–15 and zoom‐inset). A similar dark stain accumulation was also visible in nsTEM of T4 bacteriophages with the lanthanide ytterbium as present in UAZ.^[^ ^62^ ^]^ Contrarily, the lipid bilayer and RNA genome inside the viruses were well resolved (Figure 6, 15 and zoom‐inset).

The transition metal‐based PTA, STAIN 77, Nano‐W, and NanoV achieved similar negative‐staining contrast and resolution, with PTA, STAIN 77, and Nano‐W allowing the highest resolution and contrast imaging of the viral lipid bilayer and individual spike proteins of all the uranyl‐alternative stains assessed here, followed by NanoV that provided lower resolution and contrast (Figure 6, 16–27). PTA, STAIN 77, and Nano‐W also resolved well the RNA genome inside the viruses, which was less visible in NanoV (Figure 6, 18,21,24,27 and zoom‐insets). Notably, PTA repeatedly produced an irregular stain‐distribution on Formvar support grids despite hydrophilization of the Formvar film using poly‐L‐lysine (PLL)^[^ ^5^ ^]^ (Figure 6, 16–18 and zoom‐inset). Also STAIN 77 and to a lesser extent Nano‐W showed unevenly distributed stain on Formvar support grids, yet the unstained patches could be more easily avoided during imaging, especially at higher magnifications (Figure 6, 21,24 and zoom‐insets). Interestingly, the contrast and especially the resolution of the spike proteins and RNA genome were inferior with UA compared to several stain alternatives (Figure 6, 1–3).

In summary, the transition metal‐based stains PTA, STAIN 77, and Nano‐W, followed by NanoV, allowed better contrast and resolution than UA for the visualization of inactivated influenza‐A viruses, including the round and elongated shapes, spike proteins, lipid bilayer, and RNA‐genome, rendering them suitable uranyl‐replacements for viruses despite their less homogeneous stain‐distribution on Formvar (see Table 3).

### 2.5 Negative‐Staining of Inactivated Influenza‐A Viruses on Carbon Support Grids

**Figure 7:**
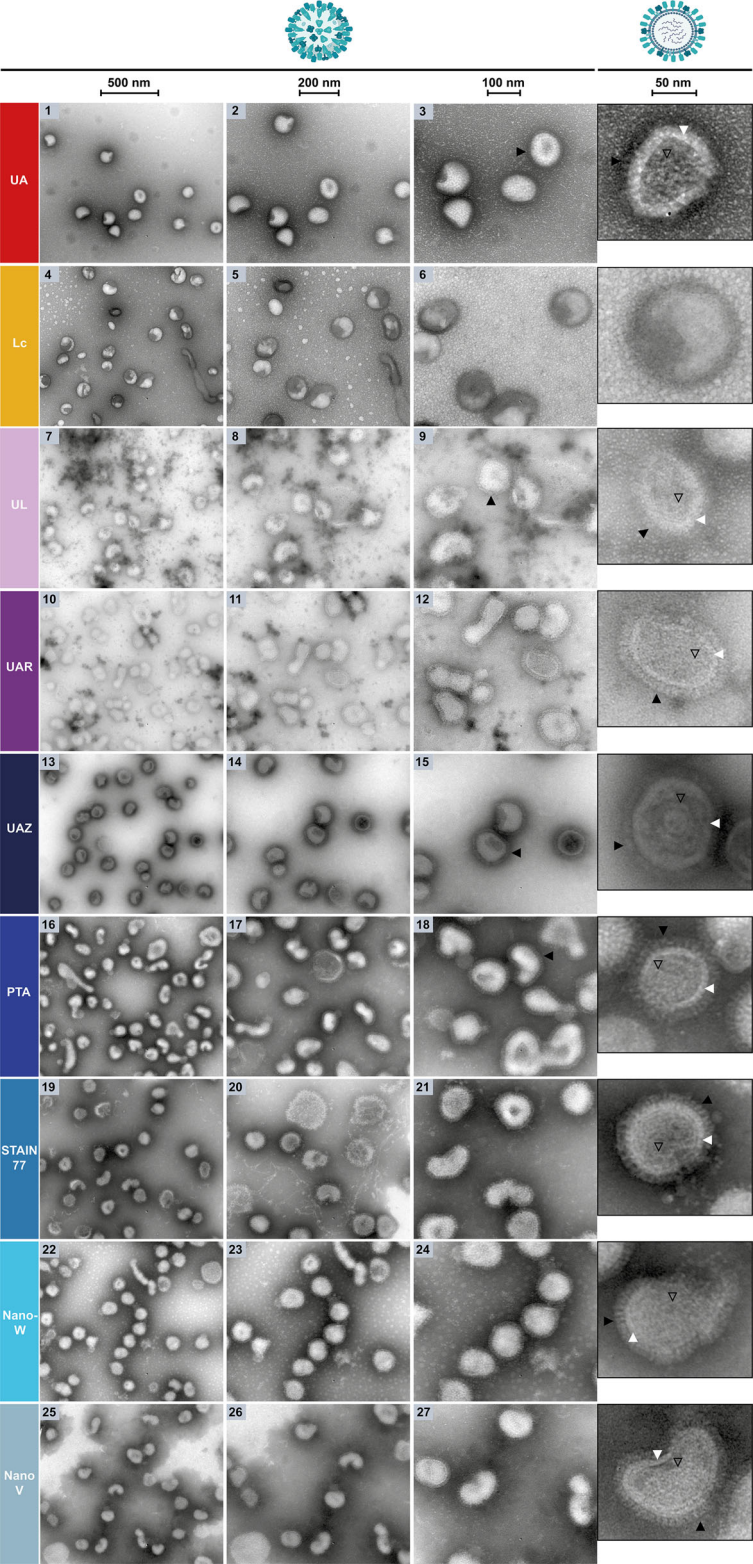
NsTEM of inactivated influenza‐A virus on carbon support grids. The three columns and zoom‐insets show the sample at increasing magnifications (see scale bars: 500, 200, 100, 50 nm). The viruses and their surface spike proteins are visible (black arrows), as well as their lipid bilayer (white arrows) and internal RNA‐genome (black outlined arrows), as schematically shown in the cartoons.

To also compare the stains' nsTEM performance on different grid support films, they were assessed and benchmarked against UA (**Figure** **7**, 1–3 and zoom‐inset) with inactivated influenza‐A virus applied to untreated carbon support grids instead of Formvar support grids hydrophilized with poly‐L‐lysine (Figure 6).

Also on carbon support grids, Lc provided contrast, negative‐staining and even stain‐distribution, but no high‐resolution characterization was possible of the spike proteins, lipid bilayer, or RNA‐genome. Moreover, Lc again showed beam damage, this time appearing as small black dots and white droplets (Figure 7, 4–6). The lanthanide‐based UL and UAR stains performed similarly as on Formvar support grids with lighter negative‐staining contrast and stain precipitates likely of gadolinium phosphate^[^ ^100^ ^]^ (Figure 7, 7–12). While higher resolution was achieved for both UL and UAR on carbon support grids, potentially due to the thinner support film of carbon compared to Formvar, a clearer visualization of the individual spike proteins and lipid bilayer was again possible with UAR, yet inferior to other stain alternatives (Figure 7, 12 and zoom‐inset). The resolution of the RNA‐genome remained unsatisfactory for both stains, although slightly better in UAR also compared to its Formvar sample (Figure 7, 9,12 and zoom‐insets).

On carbon support grids, UAZ provided stronger contrast again, but less dark stain accumulation around the viruses than on Formvar support grids, allowing higher resolution imaging of the individual spike proteins, yet still unsatisfactory compared to other stains (Figure 7, 13–15 and zoom‐inset). Also the lipid bilayer and RNA‐genome inside the viruses were better visible than on Formvar, yet less well resolved than with other stain‐alternatives (Figure 7, 15 and zoom‐inset).

PTA, STAIN 77, Nano‐W, and NanoV resulted also on carbon support grids in similar negative‐staining contrast and resolution, with STAIN 77 and Nano‐W achieving the highest resolution‐ and contrast‐imaging of the viral lipid bilayer and individual spike proteins of the UA‐alternatives assessed, followed by PTA and NanoV (Figure 7, 16–27). PTA achieved satisfying contrast and now well‐distributed staining even without hydrophilization of the carbon support grid, but lower resolution (Figure 7, 16–18). NanoV provided high resolution, but slightly lower contrast, as designed, and more uneven stain‐distribution (Figure 7, 25–27 and zoom‐inset). Nevertheless, similarly to STAIN 77, the unstained patches in NanoV were avoidable during imaging at higher magnifications (Figure 7, 19–21, 25–27 and zoom‐insets). PTA, STAIN 77, Nano‐W, and NanoV also resolved well the RNA‐genome inside the viruses (Figure 7, 18,21,24,27 and zoom‐insets). Surprisingly, UA produced more contrast on carbon than Formvar support grids but performed worse for the virus sample than the transition‐metal based PTA, STAIN 77, Nano‐W, and NanoV stains (Figure 7, 1–3 and zoom‐inset).

Taking all criteria together, tungsten‐based STAIN 77 and Nano‐W, followed by tungsten‐based PTA and vanadium‐based NanoV, allowed better visualization of influenza‐A viruses with their spike proteins, lipid bilayer, and internal RNA‐genome on carbon support grids than UA. Despite more uneven stain‐distribution of STAIN 77 (to a low extent) and NanoV (to a higher degree) than UA, high‐resolution imaging at higher magnifications was not obstructed and still superior to UA, suggesting them nonetheless as excellent uranyl‐replacements for nsTEM of viruses. Notably, for both grid types, the lanthanide‐based UAR, the transition metal‐based PTA and NanoV and the lanthanide‐transition metal hybrid UAZ satisfactorily visualized the influenza PE‐rich membrane and the liposomal POPC‐membranes discussed previously. Similarly superior performance of phosphotungstic acid (as in PTA and UAZ) and the lanthanides samarium and gadolinium (as in UAR) compared to UA was recently described by Ishii et al.^[^ ^8^ ^]^ for chromatophores containing PE‐lipids and proteins. The lanthanides samarium and gadolinium also stained viral T4 bacteriophages comparably or even superiorly to UA in nsTEM.^[^ ^62^ ^]^ Interestingly, more stains sufficiently stained POPC than PE for nsTEM in our study, likely due to its stabler, larger head with a permanent positive charge.^[^ ^101^ ^]^ In summary, the transition metal stains PTA and NanoV worked well for both membrane lipid‐compositions, the tungsten‐based STAIN 77 and Nano‐W better for PE‐lipids, viral proteins, and nucleic acids (see Table 3).

### 2.6 Negative‐Staining of Globular Iron‐Loaded Ferritin Proteins on Carbon Support Grids

**Figure 8:**
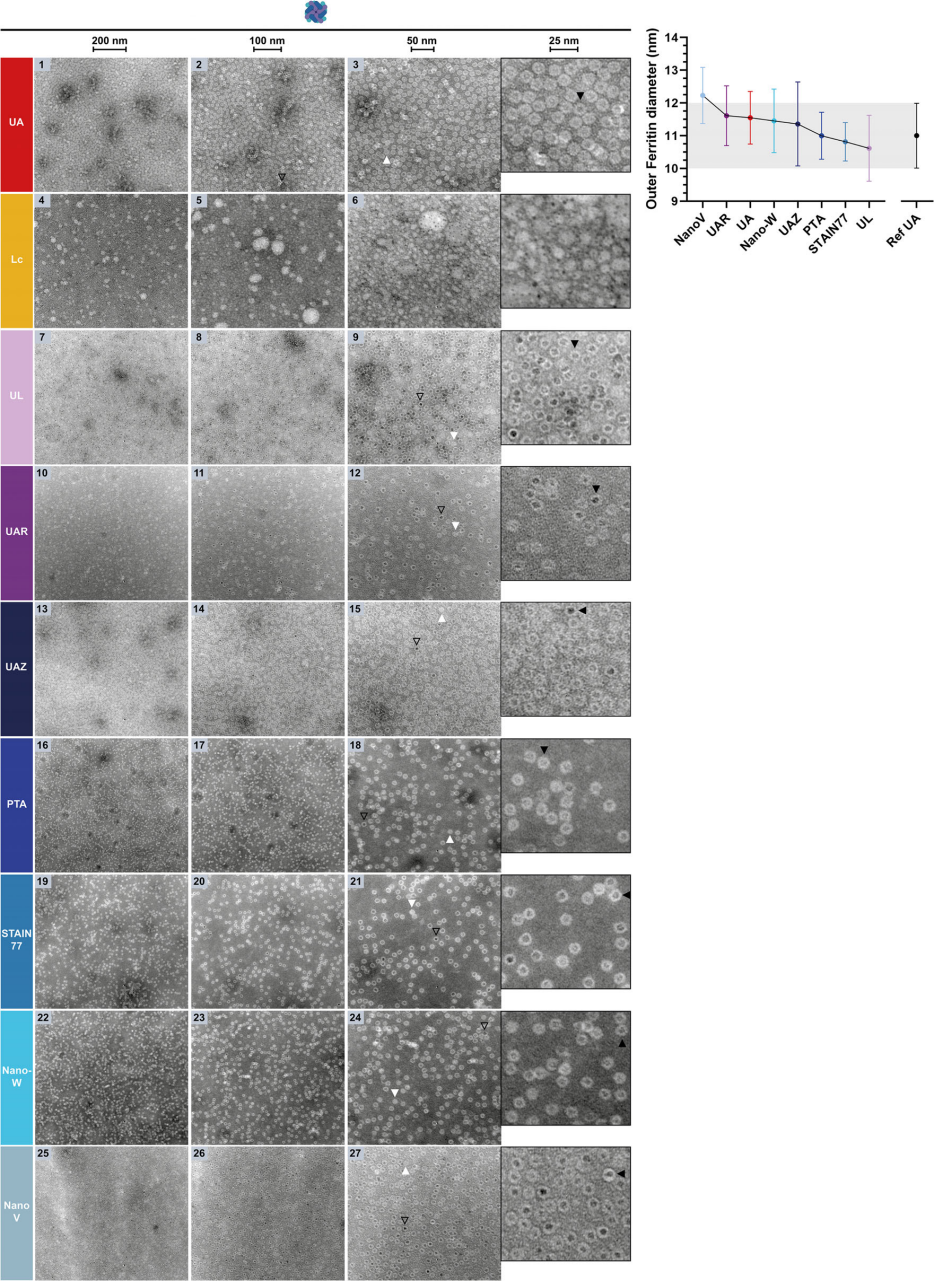
Left: NsTEM of globular horse‐spleen ferritin rings with iron‐loaded core on carbon support grids. The three columns and zoom‐insets show the sample at increasing magnifications (see scale bars: 200, 100, 50, 25 nm). Ferritin particles with their ring subunits appeared in top view (black arrows), see cartoon (not true to size for visibility). Occasionally also the iron cores (black outlined arrows) and side views (white arrows) are visible. Right: Plot of the mean outer diameters ± SD of horse‐spleen ferritin from nsTEM images as on the left. Due to inferior staining performance, ferritin diameters were unmeasurable with Lc. The mean outer diameter ± SD reported in literature for horse‐spleen ferritin from UA‐stained nsTEM micrographs is shown as grey area for reference.^[^ ^75^ ^]^

After assessing the uranyl‐alternatives on synthetic polymer and phospholipid nanoparticles as well as hybrid nanoparticles with the viruses containing proteins, lipids and nucleic acids, the stains were evaluated next against UA (**Figure** **8**, Left, 1–3 and zoom‐inset) on iron‐loaded, ring‐shaped horse‐spleen ferritin on carbon support grids as an example for globular and commonly nsTEM‐characterized proteins.^[^ ^9^, ^31^, ^96^ ^]^

Here, Lc failed to provide negative‐staining, even stain‐distribution, beam damage‐resistance, and sufficient resolution for the visualization of the ferritin rings (Figure 8, 4–6). Contrarily to the viruses (Figures 6 and 7), UL and UAR resulted in a different staining‐contrast for ferritin, with UL achieving a similar contrast‐lightness as UAZ (Figure 8, 7–15 and zoom‐insets). Notably, UL and UAR did not show any stain precipitates as with the viruses, indicating a compatibility of these gadolinium stains with phosphate‐free buffer such as MES used for ferritin in contrast to the viruses' PBS buffer^[^ ^10^ ^]^ (Figure 8, 7–12 and zoom‐insets). Regarding the resolution of the ring subunits, UL performed slightly better than UAR and UAZ. In all three stains, and especially for UL and UAR, ferritin rings with a dark iron‐loaded core were visible, i.e., the density of iron was not obscured by the negative‐staining. Such dark iron‐cores were also visible in the nsTEM images reported by Ishii et al.^[^ ^9^ ^]^ for the lanthanide dysprosium present in UL (Figure 8, 7–15 and zoom‐insets). Notably, the contrast of PTA, STAIN 77 and Nano‐W was darker, while Nano‐V resembled more UL and UAZ, also resolution‐wise (Figure 8, 16–27 and zoom‐insets). Amongst PTA, STAIN 77 and Nano‐W, STAIN 77 resolved ferritin's subunits with slightly higher resolution and showed a few more stained iron‐loaded cores, yet inferior to NanoV, which with its designed lower contrast masked the scattering contrast of the iron core to a lower extent (Figure 8, 16–27 and zoom‐insets). However, while several stains including UA showed occasionally a few darker stain spots, Nano‐V showed larger areas of unevenly lighter and darker stain (Figure 8, 16–27). Interestingly, UA achieved less dark contrast compared to PTA, STAIN 77 and Nano‐W, but almost completely obscured the iron core of ferritin, while ring subunit resolution was satisfactory, similarly to data reported by Ishii et al.^[^ ^9^ ^]^ (Figure 8, 1–3, 16–27 and zoom‐insets). For all stains, also side views of ferritin molecules besides the top‐view ring‐shape and core were visible, yet to different extents, which is relevant for nsTEM 3D reconstructions, where preferred particle orientations lead to low resolution or lacking side‐view information.^[^ ^10^, ^31^, ^64^ ^]^

Additionally, the outer diameter of ferritin particles was measured in nsTEM images as in Figure 8 (Left) to compare with the mean diameter of 11.0 ± 0.7 nm reported in literature from UA‐stained nsTEM micrographs for horse‐spleen ferritin.^[^ ^75^ ^]^ This reference diameter is in the range determined also with other methods, indicating a less pronounced size‐overestimation of UA for horse‐spleen ferritin in the reference nsTEM images.^[^ ^84^, ^97^ ^]^ In our UA‐images, a mean ferritin diameter of 11.5 ± 0.8 nm (±SD) was measured, 10.6 ± 1.0 nm in UL, 11.6 ± 0.9 nm in UAR, 11.4 ± 1.3 nm in UAZ, 11.0 ± 0.7 nm in PTA, 10.8 ± 0.6 nm in STAIN 77, 11.5 ± 1.0 nm in Nano‐W and 12.2 ± 0.9 nm in NanoV (Figure 8, Right). Contrary to PMMA size measurements, where the means of four stains were out of reference range (RR), three means on the RR border, two within RR and one (PTA) ±SD inside RR, the means of seven stains were within RR for ferritin, only one out of RR and two ±SD inside RR, indicating that all stains resulted in rather similar mean sizes. Yet, STAIN 77 and PTA reached comparatively the highest size accuracy (smallest SD) for ferritin, and UAZ produced the largest size measurement inaccuracy (±SD) for both ferritin and PMMA.

Altogether, the tungsten‐based STAIN 77 followed by PTA achieved higher accuracy of size measurements (smaller SD), better contrast and higher resolution of ferritin's iron core as well as ring subunits than UA, rendering them perfectly suitable UA‐replacements. Moreover, STAIN 77 outperformed other tungsten‐based stains for globular ferritin and viral spike proteins, despite its low tungsten content (<1% vs. PTA and Nano‐W at 2%, UAZ at 0.1–2%). This may suggest a beneficial role of the additional component lithium in STAIN 77, or better protein structure stabilization of ferritin and viral spike proteins at the higher pH of 8.2 of STAIN 77 (but not as high as pH ≈12 of Lc). Conversely, both UA and UAZ, with the unphysiologically low pH of 4–5, showed a weaker performance in the nsTEM analysis of these protein samples (see Table 3).

**Figure 9:**
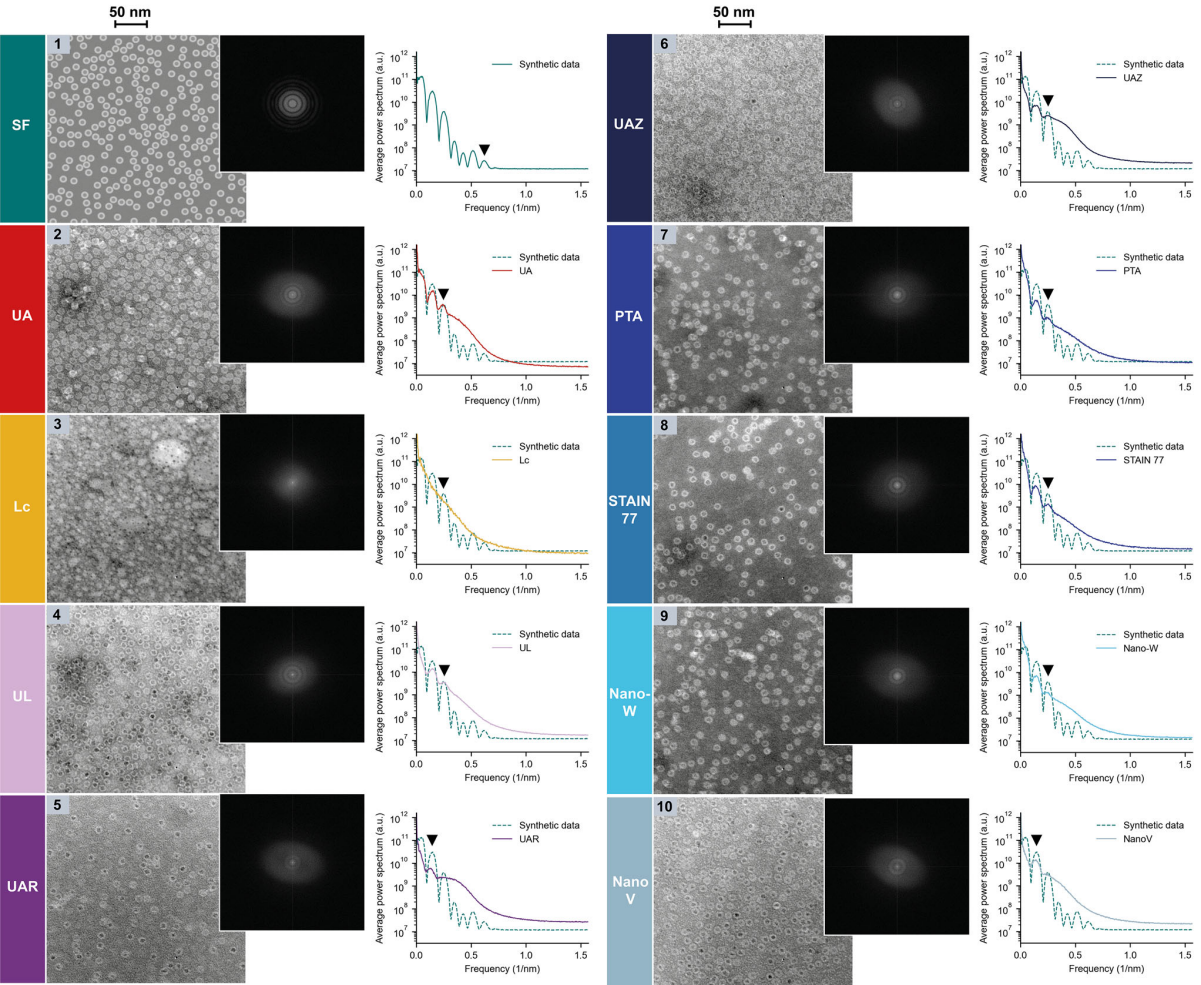
Frequency domain analysis of synthetic ferritin images (SF) and experimental nsTEM images of globular horse‐spleen ferritin rings on carbon support grids as in Figure 8. Left: Synthetic and nsTEM images at the same magnification. Middle: Logarithmic power spectrum of the corresponding image on the left. Right: Radially averaged power spectrum across several images of the same stain (one representative image shown on the left).

In addition to the evaluation of the contrast and resolution differences of the stains based on a qualitative assessment of the images and the size measurements, the highest magnification images of ferritin produced with the different stains were subjected to a frequency domain analysis and compared with synthetic data as reference to further evaluate the resolution achieved with the different stains (**Figure** **9**; Figures S1 and S2, Supporting Information). Ferritin was selected for this analysis due to its use as an organic TEM‐reference particle and its regular shape with a 432‐point symmetry and a distinct ring and core structure,^[^ ^83^ ^]^ which significantly facilitate the identification and comparison of distinct, characteristic frequency peaks in the frequency domain analysis with the synthetic data as a reference, in contrast to more complex and irregular samples such as influenza‐A viruses.

**Figure 10:**
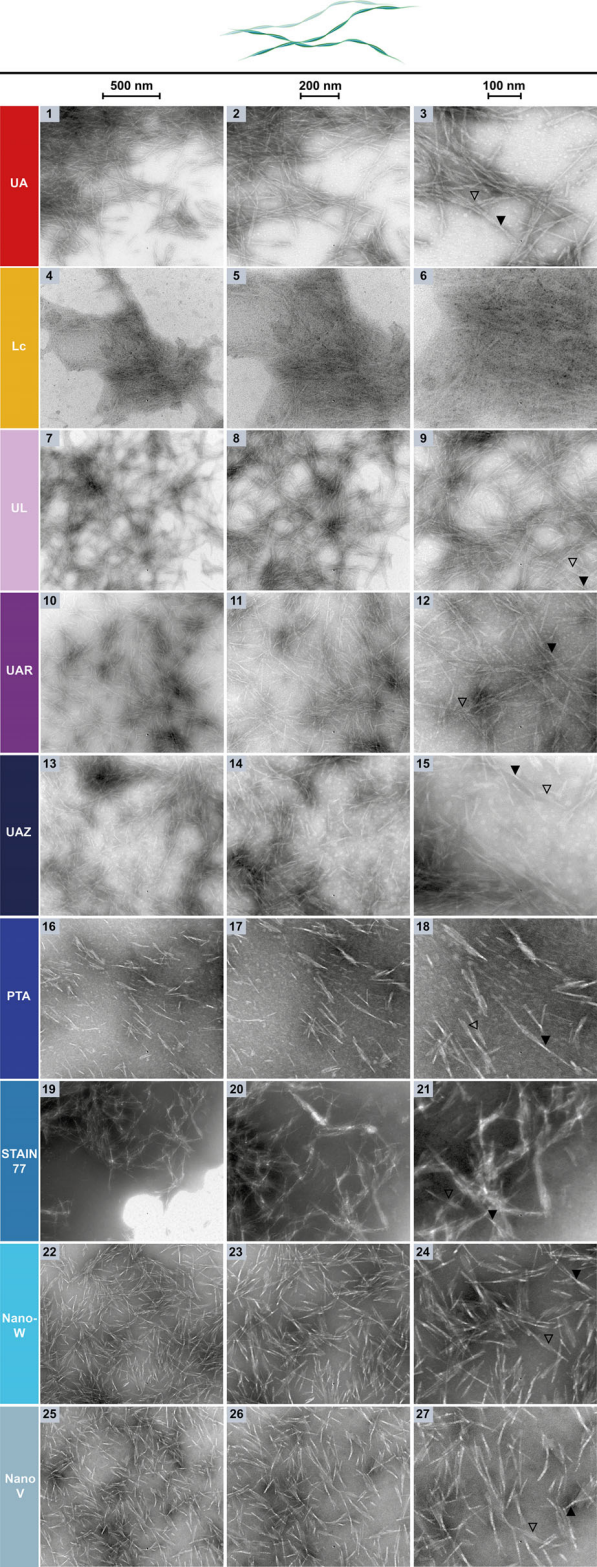
NsTEM of amyloid fibrils formed by the wildtype yeast pyruvate kinase amyloid core on carbon support grids. The three columns show the sample at increasing magnifications (see scale bars: 500, 200, 100 nm). The amyloid core fibrils, their individual protofibrils (black outlined arrows) and their typical, regularly spaced crossovers (black arrows) are visible. Cartoon is not true to size for visibility.

In order to have a clear reference for the expected spectral components of an ideal nsTEM image of ferritin, synthetic images of ferritin (SF) were generated with comparable ferritin ring size (outer diameter: 11.0 nm,^[^ ^75^ ^]^ inner diameter: 5.4 nm from our images), density and distribution on the grid and grey values as observed in UA‐stained nsTEM images. Furthermore, a lowpass filter was applied to the synthetic images and white noise was added to mimic the experimental acquisition conditions (Figure 9, 1, Figures S1 and S2, Supporting Information). As visible from the SF images, the ring and core structure of the protein gives rise to regularly spaced concentric rings in the image power spectrum (Figure 9, 1, inset), which appear as clear peaks in the radially averaged spectrum (Figure 9, 1, right). The ability of the different stains to resolve the ring structure of ferritin can then be assessed by comparing the spectral response of the corresponding images against the SF reference (Figure 9, 1‐10, inset and right plot). Images with a high ferritin ring resolution will show clear high frequency peaks associated to the ring structure of ferritin, while limited resolution will lead to peak smoothing and lack of distinct higher frequency signals (Figure 9, 1‐10, right). It can be observed that the peaks in the average power spectrum of the experimental images match those of SF from low frequencies up to a certain high frequency peak, after which they smoothly reach a baseline noise level (Figure 9, 1‐10, right). While this decreasing behavior is dictated by the acquisition parameters, the highest frequency ferritin ring peak resolvable is characteristic of the particular stain (black arrows in Figure 9, 1‐10, right). The higher the frequency of that peak, the better the ferritin ring resolution achieved with that stain. The highest frequency peak recovered in the experimental data is around 0.25 nm^−1^, which appears clearly defined in UA, UL, PTA, STAIN 77, and Nano‐W (black arrows in Figure 9, 2, 4, 7‐8, right). These stains are hence offering the best ferritin ring resolution. The same peak appears only weakly in UAZ, indicating a slightly worse performance (black arrow in Figure 9, 6, right). This is followed by UAR and NanoV, which have their highest frequency peak around 0.15 nm^−1^ (black arrows in Figure 9, 5, 10, right), and Lc was not able to sufficiently resolve the rings and does not show any distinct peak in its spectrum (Figure 9, 3, right). The results of this analysis are in line with the qualitative resolution assessment based on the visibility and sharpness of the ferritin rings in the nsTEM images. Notably, this frequency domain analysis focused on the structure of ferritin as a ring with a core in top view without considering the subunits or side views to reduce analysis complexity. The resolution assessment of ferritin in Table 3 includes the resolution of the ferritin rings in the frequency domain analysis and qualitative nsTEM image evaluation as well as the visibility and sharpness of the ferritin subunits in the nsTEM images.

### 2.7 Negative‐Staining of Amyloid Protein Fibrils of Pyruvate Kinase (PK) on Carbon Support Grids

To compare the stains' nsTEM performance also on fibrillar proteins as opposed to globular proteins, they were assessed and benchmarked against UA (**Figure** **10**, 1‐3 and zoom‐inset) on amyloid fibrils formed by the 17 amino acid‐long amyloid core of the conserved glycolytic enzyme pyruvate kinase (here from yeast^[^ ^25^ ^]^) on untreated carbon support grids. Amyloid fibrils are elongated structures, often extending up to several micrometers in length, consisting of several protofibrils twisting around each other at regular crossovers.^[^ ^25^, ^37^ ^]^ They are characterized by a cross‐β organization, which can be formed by many different proteins like beta‐amyloid,^[^ ^102^ ^]^ alpha‐synuclein^[^ ^18^, ^26^, ^27^ ^]^ or Orb2.^[^ ^103^, ^104^ ^]^ Visualizing these structures is crucial, as they are implicated in several common neurodegenerative diseases like Alzheimer's and Parkinson's, and have also recently been recognized for their essential physiological functions.^[^ ^23^, ^24^, ^25^, ^98^, ^99^ ^]^ Examining their morphology by nsTEM is particularly important to identify different polymorphs that can have distinct biological activities and pathological consequences.^[^ ^25^, ^26^, ^104^ ^]^

Importantly, the behavior of amyloids and fibrillar proteins in negative‐staining differs significantly from that of globular proteins or spherical particles. Amyloid fibrils are prone to self‐assemble and form clusters that often attract the stain via electrostatic or hydrophobic interactions, leading to uneven stain‐distribution and positive‐staining.^[^ ^24^, ^25^, ^37^ ^]^ The extent of homogenous stain‐distribution and negative‐staining as well as the stability of the fibrils to visualize their individual protofibrils and crossovers can be dependent on the pH of the buffer and staining solution as well as the hydrophobicity/ionic composition of the stain.^[^ ^10^, ^24^, ^25^, ^37^ ^]^ Indeed, we showed in previous studies that certain amyloids like those formed by PK are able to sense pH and respond to changes in intracellular pH as part of their vital cellular functions.^[^ ^25^ ^]^ Moreover, they can interact with charged molecules (e.g., metabolites) or hydrophobic buffer components (e.g., glycerol for fibril stabilization) that attract or precipitate stain components, causing stain accumulation at fibril clusters.^[^ ^10^, ^24^, ^25^, ^37^ ^]^ Based on our extensive experience with PK amyloids in nsTEM using UA,^[^ ^24^, ^25^ ^]^ this example specimen was used here.

Lc produced the typical uneven stain‐distribution, accumulating around fibril clusters and hindering the resolution of protofibril‐ or crossover details even at high magnifications (Figure 10, 4‐6 and zoom‐inset). On the contrary, UL, UAR, UAZ, PTA, Nano‐W, and NanoV surprisingly all distributed evenly on the carbon support grids, including around clustered fibrils. They preserved fibril structures well despite their differing pH levels (see Table 1), and produced high contrast and resolution, allowing UA‐comparable imaging of the protofibrils and crossovers (Figure 10, 7‐18, 22‐27, and zoom‐insets). Such a UA‐equivalent nsTEM performance of the lanthanides samarium and gadolinium present in UAR and UL was also visible in the images reported by Nakakoshi et al.^[^ ^65^ ^]^ for beta‐amyloid fibrils. Minor differences, inconsequential for the imaging results, were observed in nsTEM for UAZ and NanoV, which provided marginally less contrast than the other stains. Contrarily, STAIN 77 accumulated around fibril clusters, locally producing very dark negative‐staining, even when the carbon support film was hydrophilized with PLL, and obscuring the higher resolution details such as the individual protofibrils and clear crossover positions, similarly to Lc yet to a lower extent (Figure 10, 4–6, 19‐21 and zoom‐inset). This might be due to their higher pH of up to 8.2 (STAIN 77) and ≈12 (Lc), which could potentially initiate disassembly of the pH‐sensitive PK fibrils during the short staining period,^[^ ^24^, ^25^ ^]^ resulting in “fuzzier” fibril contours and hindering high‐resolution imaging. Additionally, UA, UL, UAR, and UAZ bordered to positive‐staining, with darker staining around fibrils and lighter staining in the background, while the transition metal‐based PTA, STAIN 77, Nano‐W and NanoV stains achieved darker staining and high negative‐staining contrast (Figure 10, 1‐3, 7‐27, and zoom‐insets).

Overall, most uranyl‐alternatives performed surprisingly well and were comparable to UA as used in our previous nsTEM‐analyses of this amyloid fibril sample.^[^ ^24^, ^25^ ^]^ Our results suggest UL, UAR, UAZ, PTA, Nano‐W, and NanoV as highly suitable uranyl‐replacements for the nsTEM characterization of fibrillar proteins like amyloids and high‐resolution imaging of their substructures such as protofibrils and crossovers. While STAIN 77, followed by PTA, performed best for nsTEM analysis of globular ferritin and viral spike proteins as discussed previously, PTA appears to achieve better imaging results also for fibrillar proteins, especially for amyloid fibrils sensitive to higher pH levels like the PK amyloid core peptide^[^ ^25^ ^]^ (see Table 3).

### 2.8 Positive‐Staining of Ultrathin Sections of Resin‐Embedded Human Cells

**Figure 11:**
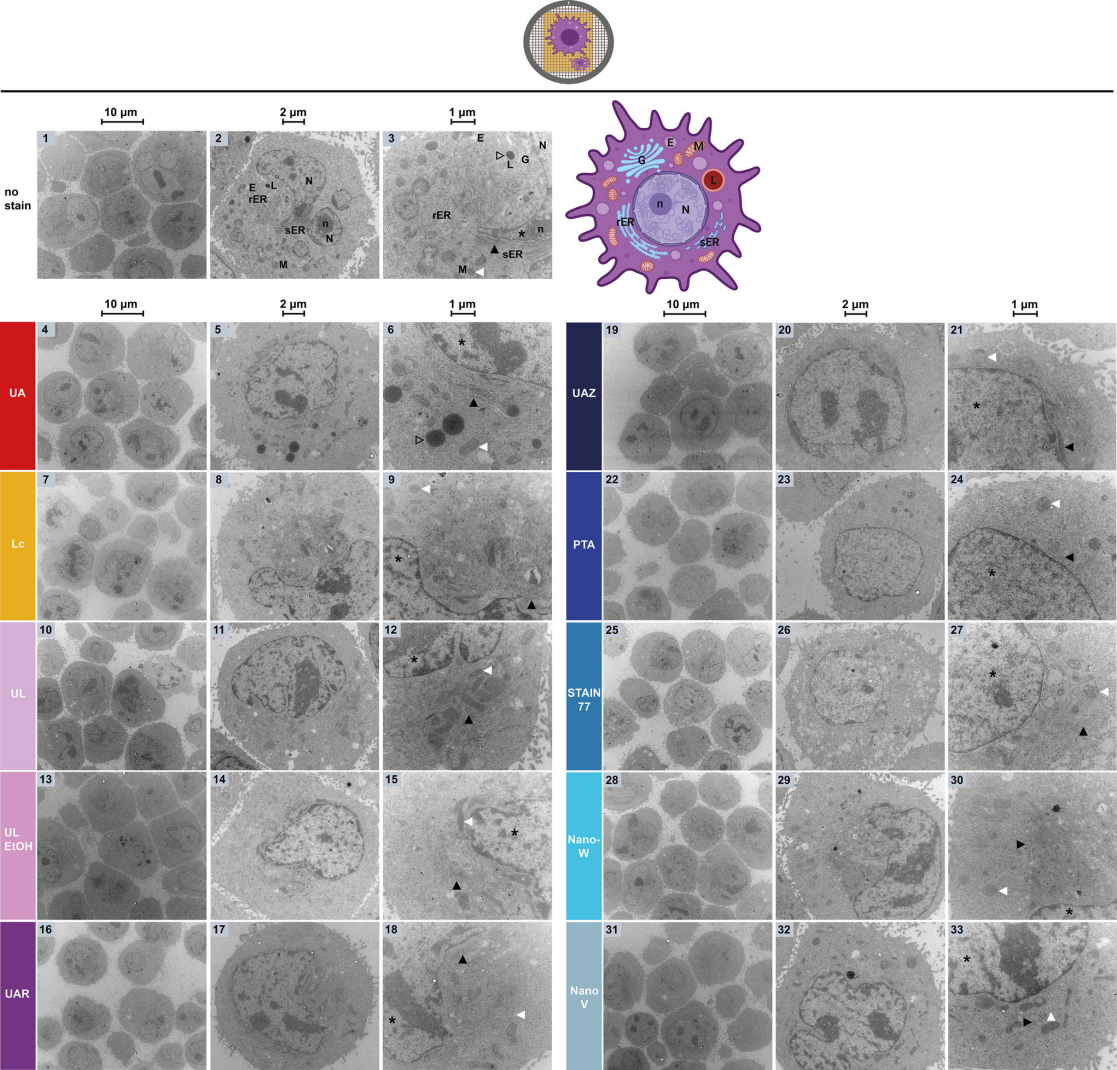
PsTEM of ultrathin epoxy sections of A549 cells on Formvar support grids with uranyl‐alternative stains benchmarked against the unstained sample and UA. The three columns show the sample at increasing magnifications (see scale bars: 10 , 2 , 1 µm). N: nucleus, n: nucleolus, M: mitochondria, E: endosomes, L: lysosomes, G: Golgi, sER: smooth endoplasmic reticulum, rER: rough endoplasmic reticulum with ribosomes. Highlighted organelles: nucleus with membrane (*), Golgi/ER membranes (black arrows), mitochondria with cristae (white arrows), and lysosomes (black outlined arrows). Cartoons not true to size for visibility.

**Figure 12:**
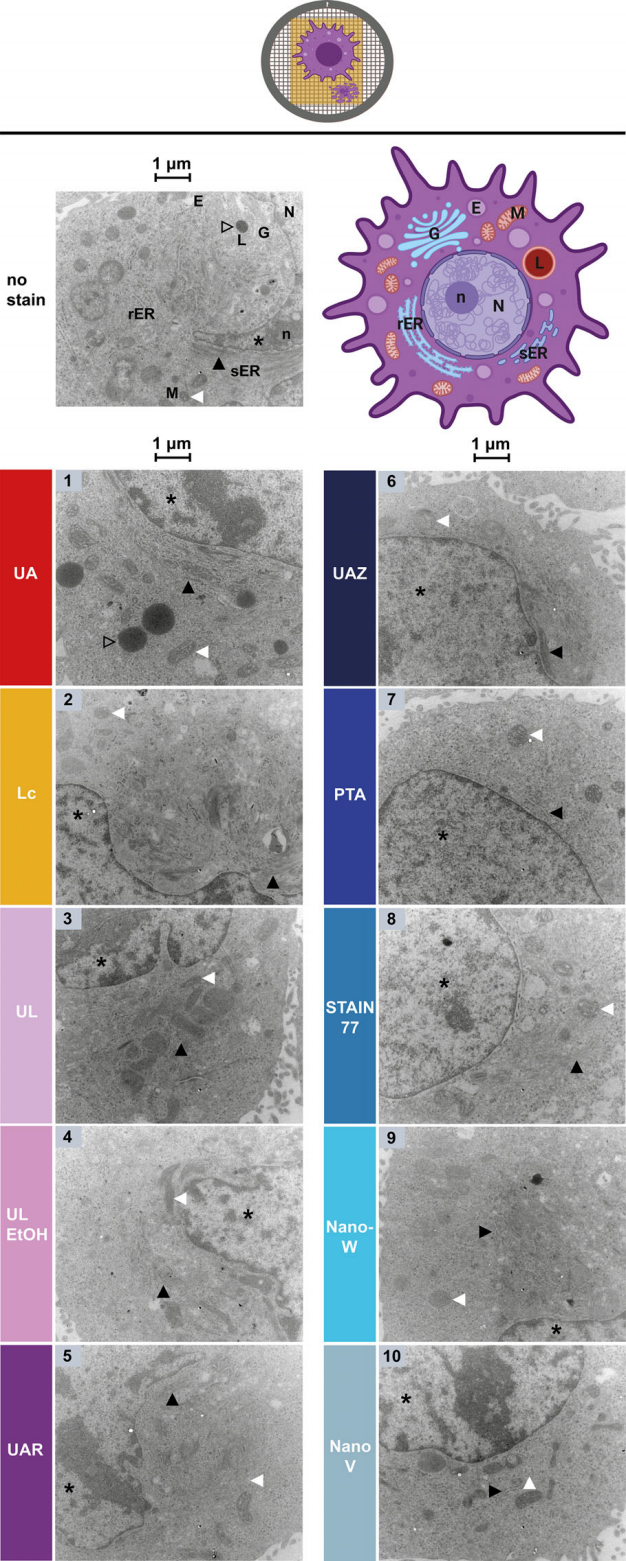
The higher magnification images of Figure 11 of psTEM of ultrathin epoxy sections of A549 cells on Formvar support grids with uranyl‐alternative stains benchmarked against the unstained sample and UA are shown here again in a larger format for better comparison. N: nucleus, n: nucleolus, M: mitochondria, E: endosomes, L: lysosomes, G: Golgi, sER: smooth endoplasmic reticulum, rER: rough endoplasmic reticulum with ribosomes. Highlighted organelles: nucleus with membrane (*), Golgi/ER membranes (black arrows), mitochondria with cristae (white arrows), and lysosomes (black outlined arrows). Scale bars: 1 µm. Cartoons not true to size for visibility.

Besides negative‐staining of organic particles, positive‐staining of cells for ultrastructural analysis is regularly employed in TEM.^[^ ^12^, ^13^, ^14^, ^15^, ^16^, ^17^, ^18^, ^19^, ^45^, ^55^ ^]^ For beam electrons to pass through the sample and create an image in TEM, the specimen needs to be ultrathin, generally below 100 nm,^[^ ^3^ ^]^ yet cells and tissues from all kingdoms of life are usually in the micrometer range.^[^ ^4^, ^12^, ^19^, ^20^, ^45^, ^50^, ^53^, ^54^, ^72^, ^74^ ^]^ They thus require ultrastructural preservation by fixation, resin embedding and sectioning into ultrathin sections of typically 30–100 nm for room‐temperature TEM.^[^ ^3^ ^]^ Cell contrast is introduced during processing through en‐bloc post‐fixation, where the entire specimen is positively stained with osmium tetroxide and UA, followed by positive post‐staining of the resin‐embedded sections, typically by single‐ or combinational double‐staining with UA and lead citrate.^[^ ^1^, ^4^, ^62^, ^65^ ^]^ Thereby, the contrast of cell membranes and cellular organelles such as the nucleus or endoplasmic reticulum (ER) (**Figure** **11**, 1‐3, schematic cartoon) can be enhanced additionally to osmium post‐fixation staining.^[^ ^65^ ^]^ Here, we compare the single post‐staining psTEM performance of the stain alternatives benchmarked against an unstained control (Figure 11, 1‐3) and UA (Figure 11, 4‐6) on ultrathin sections of epoxy‐embedded human adenocarcinoma cells (A549). While numerous organelles are visible in the ultrastructure of these cells,^[^ ^86^ ^]^ a selection is highlighted in Figure 11 for better comparability. Additionally, **Figure** **12** with the highest magnification images provides enhanced visibility and facilitates a more detailed comparison of the staining of individual structures inside the cells.

Compared to the sections without post‐staining, UA achieved darker staining, better contrast, and higher resolution, visualizing all the organelles and membranes uniformly well. Also Lc provided satisfactory contrast and resolution, as visible especially at higher magnifications with a more contrasted nucleus than in UA, the dark stained ER/Golgi and the clear mitochondrial cristae, despite lighter staining of mitochondria compared to UA (Figure 11, 4‐9, Figure 12, 1‐2). Lc induced only minimal damage to the Formvar film or epoxy sections, which altogether is contrary to the insufficient nsTEM performance of Lc for all organic nanoparticles in this study. Likely the epon surrounding the cells in the sections protected them against the beam damage and structural artifacts from the high pH of Lc (≈12) observed for the particle samples directly exposed to the stain. Nonetheless, stain precipitates were more abundant for Lc than for other stains also on the sections, despite keeping Lc at its stable high pH and under minimal air‐exposure.^[^ ^59^ ^]^

Aqueous UL achieved even darker stained cells and stronger contrast, as visible at the overview magnification and mitochondria, respectively (Figure 11, 10‐12). The mitochondrial cristae were better stained with aqueous UL than with Lc but inferior to UA, while the ER/Golgi membranes were less stained with aqueous UL than both Lc and UA (Figure 12, 3). UL preserved the Formvar film and sections, yet showed some stain precipitates, and its resolution was lower than in UA and Lc. Interestingly, UL in ethanol provided less contrast and much lighter staining, as obvious particularly at higher magnifications for ER/Golgi membranes and the hardly visible mitochondria with cristae (Figure 11, 13‐15, Figure 12, 4). Damage of the Formvar film and large holes in the sections hinted towards an etching of the epon at the section surface by the ethanol‐dissolved stain, however, without majorly enhancing the staining. UAR achieved UA‐similar stain darkness and contrast in the overview magnification (Figure 11, 16‐18), but at higher magnification, a darker stain with lower contrast became evident, especially at the dark cytosol but lighter nucleus and mitochondria (Figure 12, 5). While the ER/Golgi membranes were difficult to resolve with UAR, the mitochondrial cristae were well contrasted and sharp (Figure 12, 5). Moreover, UAR produced numerous small holes on the sections, while leaving the Formvar film intact.

UAZ achieved even darker stain, but less contrast at low magnification overviews, similar to UL in ethanol (Figure 11, 19‐21). Interestingly, however, a UA‐comparable staining darkness was visible at higher magnifications, allowing high resolution imaging of the nuclear and ER/Golgi membranes (Figure 12, 6). In contrast to UA, UAZ stained mitochondria and their cristae much less, rendering them hardly observable (Figure 12, 6). Preferential staining of certain sample components was also observed for ultrathin sections of other cells with ytterbium‐ and PTA‐containing stains like UAZ, which allowed to discern the contraction state of muscle fibers contrarily to the uniform staining of UA.^[^ ^58^ ^]^ UAZ staining induced small cracks and folds in the sections, while PTA preserved the Formvar film and sections. Moreover, PTA achieved a UA‐comparable or superior staining darkness, contrast and resolution with sharp nuclear and ER/Golgi membranes, and well‐stained mitochondria with cristae (Figure 11, 22‐24, Figure 7, 12, 7).

STAIN 77 provided even stronger contrast than UA as visible in the lighter background of the low magnification overview (Figure 11, 25‐27). Although producing a lighter cell staining than UA evident at higher magnifications, STAIN 77 UA‐comparably or superiorly resolved the ultrastructure with sharp nuclear and ER/Golgi membranes, chromatin, and mitochondria with clear cristae (Figure 12, 8). STAIN 77 occasionally damaged the Formvar film and sections, and produced precipitates, which could, however, be diluted away with water. Nano‐W preserved the Formvar film and sections, and achieved a similar stain darkness as STAIN 77, but less contrast and resolution of the nuclear and ER/Golgi membranes, and especially the mitochondria with cristae (Figure 11, 28‐30, Figure 12, 9). NanoV provided similar stain darkness, but less contrast at low magnifications (Figure 11, 31‐33). At higher magnifications, it achieved similar contrast, staining darkness, and high resolution of the nuclear and ER/Golgi membranes, and mitochondria with cristae as UA and PTA (Figure 12, 10). NanoV also maintained intact Formvar film and sections during staining. Notably, for all stains, areas with several cells could be imaged at low and higher magnification without precipitates, folds or section holes obscuring the cells or their ultrastructure.

In summary, the tungsten‐based STAIN 77 and PTA stains, followed by NanoV, Lc and aqueous UL, produced UA‐comparable negative‐staining, contrast and resolution of the nuclear and ER/Golgi membranes, chromatin and mitochondria with cristae. Yet, all stains assessed here achieved sufficient staining for ultrastructural nsTEM of cells and organelle visualization, and preferential staining of certain organelles could be leveraged to discern specific structures in some samples^[^ ^58^ ^]^ (see Table 3).

## 3 Discussion

This study aimed to systematically assess the ns/psTEM performance of eight commercial ready‐to‐use UA‐alternative stains with lower toxicity and no radioactivity. They were evaluated regarding their ns/psTEM‐contrast, resolution, stain‐distribution, ease‐of‐use, and when applicable, size measurement accuracy, on a broad variety and complexity of samples with intrinsically low scattering contrast and different sizes, shapes, and compositions. We provide a fast and safe state‐of‐the‐art staining protocol with common ns/psTEM equipment as a suitable starting point for ns/psTEM users with different levels of experience to further fine‐tune also for specific samples of interest not assessed here and for diagnostic procedures in clinical settings.

We found that for each sample type analyzed, at least one of the UA‐alternative stains assessed in this study provided UA‐comparable or even superior negative‐/positive‐staining, allowing a nanometer‐resolution TEM characterization without compromises in imaging quality, speed, or ease‐of‐use of the staining protocol (Table 3). We further discovered that several UA‐alternatives, especially with transition metals or higher lanthanide concentration, performed UA‐comparably or superiorly for several samples, suggesting them as suitable UA‐replacements for many samples of interest of ns/psTEM users in research and clinics. In accordance, also in ns/psTEM data of previous studies, it is visible that UA is in fact not the best performing stain for all samples compared to less toxic stains.^[^ ^8^, ^9^, ^58^, ^62^ ^]^

Importantly, besides the more known PTA stain,^[^ ^5^ ^]^ we identified another, recently released tungsten‐based stain ‒ STAIN 77 ‒ as a promising universal UA‐alternative with over‐average ns/psTEM performance for many different samples. Their excellent staining properties could be explained with the higher atomic number of tungsten (74) ‒ a high z‐element similar to uranium (92) and lead (82) ‒ compared to the lanthanides of some assessed stains with a lower atomic number between 62 and 70.^[^ ^8^, ^9^ ^]^ The observed differences in ns/psTEM performance among the tungsten‐based stains could originate from the chemical form they adopt in the stain solution (cationic, anionic, coordinated, etc.^[^ ^8^, ^65^ ^]^) and their interaction with additional stain components, which could provide more uniform stain‐distribution and binding to sample components, or higher stability in storage and the electron beam.

Interestingly, the tungsten‐based stains outperformed UA and Lc for many samples, likely due to the extreme pH levels of UA and Lc leading to strong interactions with the sample, and resulting in structural artifacts, stain‐accumulation, particle size overestimation, and lower resolution imaging.^[^ ^5^, ^8^, ^10^, ^20^, ^38^, ^64^, ^66^ ^]^ While producing satisfactory staining results in psTEM of cell sections, Lc was found highly unsuitable for nsTEM with the samples assessed here. In contrast, the evaluated tungsten‐based stains were more stable in the electron beam, bound more evenly to different sample components and stain precipitations were water‐removable. The superior performance of several uranyl‐alternatives in single post‐staining of cell sections suggests that apart from UA also the use of Lc could be substituted with less toxic, non‐heavy‐metal‐based stains for psTEM. A combinational application as for UA and Lc could be further investigated for the commercial stains in ns/psTEM, en‐bloc staining, or embedding with different samples and resins to potentially also replace the toxic osmium.^[^ ^58^ ^]^

Ready‐to‐use lanthanide‐stains evaluated here produced inferior ns/psTEM results, in line with previously reported low and more unspecific staining.^[^ ^58^ ^]^ Additionally, we also observed unremovable stain precipitates with phosphate‐buffers for our samples. Notably, recent research showed that other lanthanide salts and combinations produce satisfactory staining results on specific samples.^[^ ^58^, ^62^, ^65^ ^]^ The performance of some stains assessed here might be improved for certain samples, for instance by selecting a more suitable grid type (e.g., carbon over Formvar support film, see (C) in Table 3) to improve stain‐distribution or resolution, stronger hydrophilization of the grids via longer PLL incubation or glow discharging (if available) to improve sample‐ and stain‐distribution,^[^ ^10^ ^]^ or by prolonging the stain incubation time to enhance lighter staining, e.g. for UAZ and NanoV.^[^ ^58^ ^]^ Moreover, the stain and sample could be mixed and then applied to the grid, which can lead to different staining results.^[^ ^38^, ^64^ ^]^

To avoid stain precipitation, water or low‐salt buffer washes could be introduced before staining,^[^ ^10^ ^]^ or the stain concentration could be varied,^[^ ^58^ ^]^ as we found a 1:1 dilution of STAIN 77 in water reduced section damage and stain precipitates while maintaining staining contrast and resolution for sections. Furthermore, as for UA and UF, also for the gadolinium‐based stains UL and UAR the samples should be diluted in a phosphate‐free, non‐metal‐coordinating buffer such as Tris like for PK or MES as for ferritin used here to avoid the formation of insoluble precipitates.^[^ ^10^, ^100^ ^]^ MES buffer has also been suggested for ytterbium‐ and PTA‐containing stains like UAZ,^[^ ^58^ ^]^ while ethanol‐dissolved stains were reported to produce sample damage and lighter stains^[^ ^58^ ^]^ like UAZ and UL here. Another factor affecting staining performance is the pH required for stain stability, which may be too low as e.g. with UA and UAZ, or too high for a certain sample, as e.g. with STAIN 77 and Lc for PK fibrils, and lead to insufficient staining or sample damage.^[^ ^10^ ^]^ This is especially evident when stains possess slower or absent fixation properties, and the stain solution and sample interact for a longer time until air‐dried.^[^ ^65^ ^]^ As UA and UF precipitate at physiological pH, a uranyl‐alternative stain stable at such a pH, as many of the stains assessed here, is recommended.^[^ ^10^, ^65^ ^]^ Additionally, while UA occurs in a cationic form in the aqueous staining solution, other stains may be anionic, e.g. tungstate, which could influence their binding to and thus staining efficiency of specific charged sample components in ns/psTEM.^[^ ^9^, ^10^, ^24^, ^65^ ^]^

Notably, the variety of available staining solutions has been growing recently, including novel tungsten‐stains based on Preyssler‐type PTA, rather than the often commercially used Keggin‐type PTA, showing enhanced staining results.^[^ ^11^ ^]^ This facilitates finding a suitable UA‐alternative also for other techniques combined with EM, e.g. elemental analysis via energy‐dispersive X‐ray (EDX) spectroscopy to identify lanthanide‐nanoparticles in biological specimens without their EDX‐signature overlapping with the stain elements as with lanthanide stains. Such promising stain candidates should be made commercially available in the near future in a ready‐to‐use form, ideally as dispenser bottles allowing a safe and clean use with minimal waste and exposure, to facilitate a more widespread use, high reproducibility and low batch variability, which are essential in research laboratories, industry as well as in clinical diagnostics.

## 4 Conclusion

Based on our extensive screening with a standardized workflow and various organic samples, we were able to evaluate, qualify and relatively rank the ns/psTEM performance of eight UA‐alternative stains. Taken together, our key findings demonstrate a reduced necessity of UA in ns/psTEM and encourage a more widespread use of alternative stains that are safer for the users and the environment. Thus, this work represents a significant advancement and important milestone on the path to UA‐replacement, allowing also the ns/psTEM community to shift to safer and greener chemistry.

In order to support ns/psTEM users in their transition to UA‐alternative stains, we developed the multi‐dimensional, colorblind‐safe platform *GUIDE4U: Guided Utilization and Informed Decision Enabler for Uranyl‐replacement* (**Table** **3**), summarizing our findings for users of all ns/psTEM experience levels to save optimization time and easily select the ideal (green, no pattern) or sufficient (orange, downward striped) uranyl‐replacement stains for their samples, coupled with an efficient staining protocol ensuring high‐quality imaging results.

**Table 3:** Multi‐dimensional, color/pattern‐coded *GUIDE4U*: *Guided Utilization and Informed Decision Enabler for Uranyl‐replacement* platform for ns/psTEM users to select the optimal uranyl‐replacement stains for common sample types.

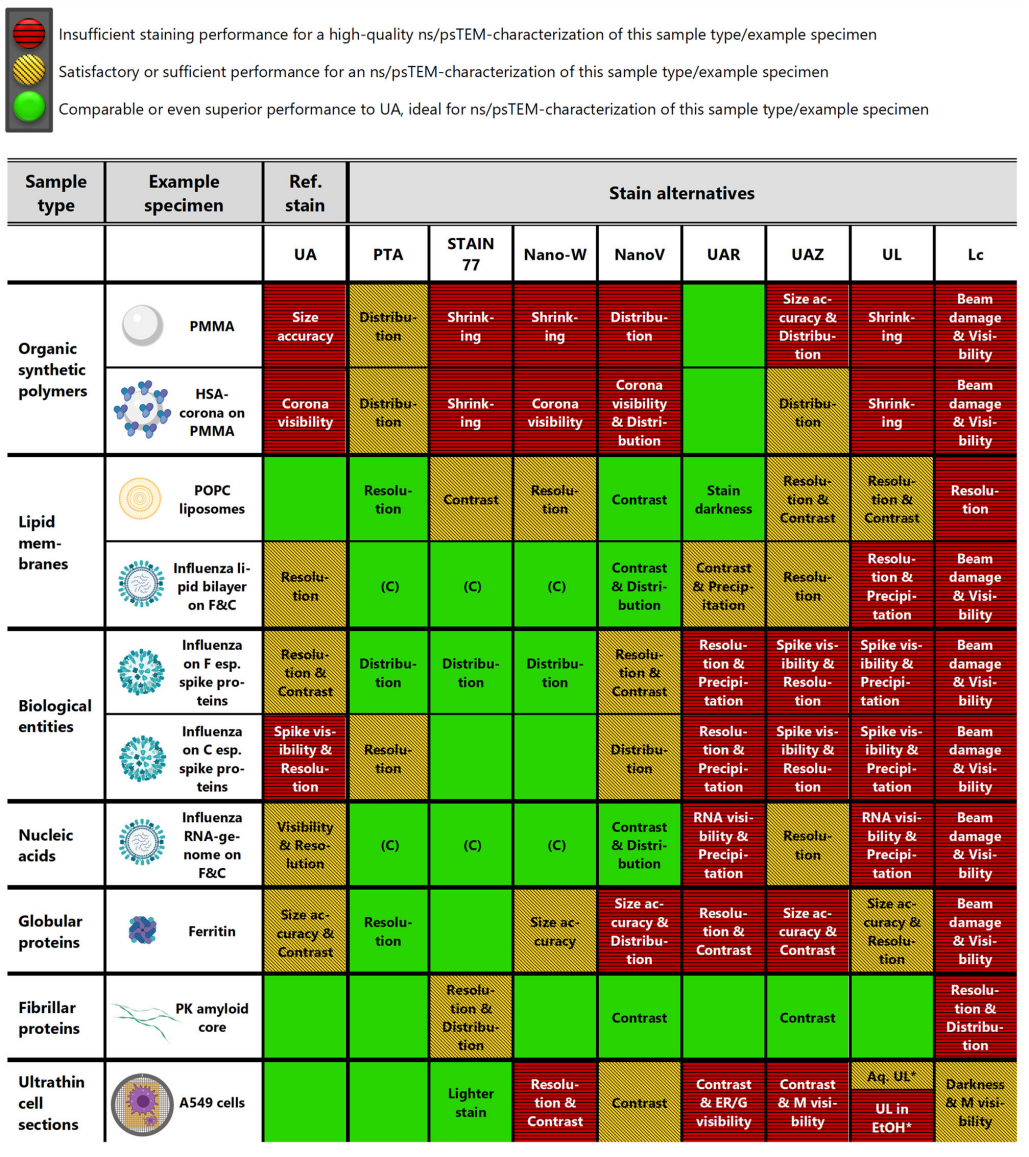

The stains are ranked relatively to each other and the reference (Ref.) UA from left to right based on their ns/psTEM performance regarding contrast, resolution, stain‐distribution, ease‐of‐use and when applicable, size measurement accuracy. Results on Formvar (F) and carbon (C) support grids were cumulatively ranked per stain, except where stated. Aqueous (Aq.) UL*: lower resolution, less ER/Golgi but stronger mitochondria (M) staining. UL in EtOH*: low contrast, stain‐darkness and limited mitochondria visibility. The resolution assessment of ferritin includes the resolution of the ferritin rings in the frequency domain analysis and qualitative nsTEM image evaluation as well as the visibility and sharpness of the ferritin subunits in the nsTEM images. Esp.: especially. Assessment criteria are mentioned in the fields if they were inferiorly fulfilled by a stain compared to the others, in green fields this marginal inferiority was inconsequential for the imaging results or could be averted with a simple protocol change.

## 5 Experimental Section

### Negative‐/Positive‐Stains

The following uranyl‐alternative stains were ordered until May 2024 and assessed in this study:

- Aqueous 2% uranyl acetate, provided by ScopeM of ETH Zurich, prepared from Uranyl acetate dihydrate (Fluka Chemika, 73 943)
- Aqueous <3% lead citrate from Electron Microscopy Sciences (EMS, SKU: 22 410, safety documentation from em‐grade)
- Aqueous 3% UranyLess Uranyl Acetate Substitute for Electron Microscopy from Electron Microscopy Sciences (EMS, SKU: 22 409)
- UranyLess <1% in 30% Ethanol from Electron Microscopy Sciences (EMS, SKU: 11000–30, safety documentation from Delta Microscopies)
- Aqueous 15% UAR‐EMS Uranyl Acetate Replacement Stain from Electron Microscopy Sciences (SKU: 22 405)
- UA‐Zero non‐radioactive EM Stain (0.1–2%) in 20% Ethanol from Agar Scientific (AGR1000)
- Aqueous 2% phosphotungstic acid pH 7.5, pH‐adjusted from aqueous 3% phosphotungstic acid pH 2.5 from Electron Microscopy Sciences (SKU: 19502–3)
- Aqueous <1% STAIN77 EM stain solution from em‐grade (SKU: GC301078‐30)
- Aqueous 2% Nano‐W^TM^ from Nanoprobes, obtained from Hölzel Biotech (NAN‐2018‐5 mL)
- Aqueous 2% NanoVan^TM^ from Nanoprobes, obtained from Hölzel Biotech (NAN‐2011‐5 mL)

For optimal staining performance, the stains were stored at the conditions as specified by the manufacturers, e.g. in the dark or at lower temperatures to avoid precipitation or degradation.

### Sample Preparation—Polymethylmethacrylate (PMMA)‐Nanoplastics with/without Human Serum Albumin (HSA)

PMMA plastics nanoparticles of 0.105 ± 0.005 µM diameter were obtained as 5% (wt) stock in water from microparticles GmbH (PMMA‐R‐0.1, batch PMMA‐R‐KM572) and stored at 4 °C. For TEM, the stock was diluted 1:100 (v/v) in MilliQ water and also stored at 4 °C until negative‐staining.

For protein corona formation on PMMA nanoplastic with HSA, a 10 mg mL^−1^ HSA stock solution (Sigma–Aldrich, SRP6182) was diluted 1:20 (v/v) in MES buffer (Sigma–Aldrich, M1317) and stored at 4 °C. For TEM, the 1:100 (v/v) PMMA stock in MilliQ water and 1:20 (v/v) HSA stock in MES buffer were mixed 1:1 (v/v), incubated for 24 h at 37 °C and shaking at 260 rpm, and then stored at room temperature until negative‐staining.

### Sample Preparation—Phosphatidylcholine (POPC) Liposomes

16:0‐18:1 PC Avanti Polar lipids were obtained as 25 mg powder from Sigma–Aldrich (850457P Avanti), diluted in 1 mL Ethanol (ACS reagent, Sigma–Aldrich, 02870–2.5L‐F), dried again at room temperature by evaporation and resuspended in 600 µL MES (Sigma–Aldrich, M1317) to form liposomes. This stock was stored at 4 °C. For TEM, the dispersion was then diluted 1:1 (v/v) in MES buffer (Sigma–Aldrich, M1317) and also stored at 4 °C until negative‐staining.

### Sample Preparation—Inactivated Influenza‐A Virus

Monovalent influenza virus A/Brisbane/59/2007 (H1N1), which was chemically inactivated and purified with a sucrose gradient to GMP grade and a hemagglutinin (HA) concentration of 1.6 mg mL^−1^, was obtained in 40% sucrose in PBS buffer from Seqirus (Melbourne, Australia) and stored at 4 °C.^[^ ^94^ ^]^ The chemical inactivation preserved the antigenic structure of the virus, while allowing a safe stain evaluation. For TEM, a 1:20 (v/v) dilution was prepared in Dulbecco's sterile‐filtered Phosphate Buffered Saline Modified 1x (PBS) w/o calcium chloride and magnesium chloride (Sigma–Aldrich, D8537) and also stored at 4 °C until negative‐staining.

### Sample Preparation—Horse‐Spleen Ferritin

Horse‐spleen ferritin type I of 50–75 mg mL^−1^ in 0.15 M sodium chloride solution with H subunits of ≈21 kDa, L subunits of ≈19 kDa and rings of 24 subunits with a core filled with up to 4500 Fe^3+^ ions was purchased from Sigma–Aldrich (F4503) and stored at 4 °C. For TEM, a 1:120 (v/v) dilution was prepared in 50 mM MES buffer (prepared from Sigma–Aldrich, M1317) and also stored at 4 °C until negative‐staining.

### Sample Preparation—Amyloid Fibrils Formed by the Yeast Pyruvate Kinase Amyloid Core

The peptide encompassing the amyloid core of yeast pyruvate kinase (Cdc19, amino acid sequence: ^376^TSTTETVAASAVAAVFE^392^) as defined in Cereghetti et al.^[^ ^25^ ^]^ was obtained from GL Biochem as lyophilized powder, resuspended to 10 mg mL^−1^ in DMSO and 10% formic acid, and stored at −20 °C. The Cdc19 amyloid core peptide was then incubated at 2 mg mL^−1^ in Tris‐HCl buffer (100 mM Tris‐HCl, 200 mM NaCl, 1 mM MgCl2) at pH 5.8 and 30 °C for 2 days to form amyloid fibrils, as described in detail in Cereghetti et al.^[^ ^25^ ^]^ For TEM, the fibril stock was diluted 1:10 (v/v) in Tris‐HCl buffer pH 5.8 and stored at room temperature until negative‐staining. CF300‐CU carbon‐coated copper grids (Electron Microscopy Sciences) were used for this sample to be able to compare the staining results with Cereghetti et al.^[^ ^25^ ^]^

### Sample Preparation—Ultrathin Epoxy Sections of Adenocarcinomic Human Alveolar Basal Epithelial Cells (A549)

The A549 cell line (CCL‐185, ATCC) was prepared for TEM as described in detail in Hendriks et al.^[^ ^51^ ^]^ for the untreated control. In short, the adherent cells were seeded to a density of 2.67 × 10^6^ in 5 ml RPMI‐1640 medium (Sigma–Aldrich, R0883) with 10% fetal bovine serum (Sigma–Aldrich, F9665), 1% penicillin–streptomycin (Sigma–Aldrich, P4458) and 0.2 mg mL^−1^ L‐glutamine (Sigma–Aldrich, G7513) in a T25 flask. After 28 h in culture at 37 °C, the cells were washed with Dulbecco's sterile‐filtered Phosphate Buffered Saline Modified 1x (PBS) w/o calcium chloride and magnesium chloride (Sigma–Aldrich, D8537), trypsinized and collected as a pellet that was then again washed two times with PBS. The cell pellet was then fixed for 2 h using 2.5% Glutaraldehyde (Sigma–Aldrich, G7651) in 0.1 M Na‐cacodylate buffer pH 7.4 (Electron Microscopy Sciences, 11 650), washed two times with 0.1 M Na‐cacodylate buffer and stored in fresh 0.1 M Na‐cacodylate buffer at 4 °C for several days. After staining the cell pellet with 1% osmium tetroxide (Electron Microscopy Sciences, 19 150) in 0.1 M Na‐cacodylate buffer in the dark at room temperature for 1 h, it was washed three times using MilliQ water. The cell pellet was subsequently dehydrated via an ethanol series (30%, 50%, 70%, 90%, 100% Honeywell Riedel‐de‐Haën Ethanol, ThermoFisher Scientific, 02860–2.5l), kept in 1 : 1 ethanol 100% and epon 812 substitute (Sigma–Aldrich, Epoxy embedding kit 45 359) for 1 h and stored overnight in 100% epon. On the following day, the cell pellet was embedded in molds using fresh epon and cured in the oven at 60 °C for 4 days. Ultrathin sections of 70 nm thickness were cut with an ultramicrotome (Leica EM UC6) from the resin blocks using an ultra 35° diamond knife (DiATOME), picked up from the diamond water boat and cast onto untreated hexagonal Formvar‐coated copper grids (100 mesh, EM resolutions, F100HxCu) using a Perfect Loop (DiATOME), and air‐dried until positive‐staining.

### Negative‐Staining and Transmission Electron Microscopy of Particle Samples

Larger particles such as viruses are often applied on Formvar support grids, which can provide a thicker and more stable support film than carbon support grids. Thus, the nsTEM‐performance of the stains for Influenza virus was compared on carbon and Formvar support grids in this study, while carbon support grids were applied for all other, smaller example specimens of non‐viral origin, and Formvar support grids only were used for the larger, heavier ultrathin sections of A549 cells (see later section). Accordingly, for TEM of Influenza virus on Formvar support grids, hexagonal Formvar‐coated copper grids (100 mesh, EM resolutions, F100HxCu) were incubated on a ≈300 µL droplet of 0.1% (w/v) poly‐L‐lysine solution (PLL, P8920, Sigma–Aldrich) for 5 min and subsequently blotted perpendicularly on Whatman filter paper before sample drop‐casting. For the other samples, untreated carbon support grids (200 mesh, EM resolutions, C200, for pyruvate kinase: CF300‐CU, Electron Microscopy Sciences) were used such that no additional equipment such as a glow discharger was necessary to provide a simple staining workflow that can be recreated in most laboratories.

Of all organic particles assessed, 5 µL sample solution/dispersion was drop‐casted and well distributed using a pipette on the PLL‐coated Formvar support grid or the untreated carbon support grid. Incubating the sample solution/dispersion on the grid instead of vice versa allowed the use of a lower sample volume and a higher sedimentation of the sample particles on the grid (especially important for fibrils with low adsorption on grids). After sample incubation on the grid for 1 min at room temperature, excess sample solution was blotted away by holding the grid perpendicularly onto the Whatman filter paper. For highly concentrated samples or particles in high salt buffers, washes with low salt buffers or water can be applied before blotting to lower particle density on the grid or decrease salt precipitation with the subsequent staining solution. Subsequently, the grid was laid onto a ≈100‐200 µl droplet of stain applied on a layer of parafilm from the ready‐to‐use stain bottle as supplied by the manufacturer, except for UAR, which had to be retrieved from its septum‐sealed vial with a needle and syringe, and PTA (no dispenser on bottle), of which 5 µl staining solution was pipetted onto the grids and well distributed using a pipette. The different grids prepared from the same sample solution were incubated with the different stains for 1 min at room temperature, then blotted perpendicularly on Whatman paper and air‐dried. Several grids were prepared per sample/stain, and representative images are shown in the Results section.

In order to ensure comparability across all example specimens, the stains were compared using the same particle solutions/dispersions and a consistent staining protocol, including equal sample incubation time on the grid and negative‐staining time. TEM micrographs were acquired using a Zeiss EM 900 standard TEM (Carl Zeiss Microscopy GmbH, Germany) with at 80 kV and different magnifications with constant hardware and software settings, including a 1k wide‐angle dual speed slow‐scan CCD camera by TRS (Series nr. 295/07, Type WIA‐7888‐V), an illumination time of 1s, equal brightness as judged from the histogram with an emission of 4 µA on all samples, a 400 µm condenser aperture, a 30 µm objective aperture, identical lenses, and a constant defocus range for all images between 50 and 52% of the maximum defocus (this range is specific to this TEM). Keeping these settings as constant as possible ensured the high comparability required for the best possible comparison to properly assess the stains' performances.

### Positive‐Staining and Transmission Electron Microscopy of Ultrathin Cell Sections

Ultrathin sections of A549 cells were generated from the same epoxy block and drop‐casted onto untreated hexagonal Formvar support copper grids (100 mesh, EM resolutions, F100HxCu) as described above. For positive post‐staining, the grids were laid onto a ≈100–200 µL droplet of stain with the sections contacting the stain solution applied on a layer of parafilm from the ready‐to‐use stain bottle as supplied by the manufacturer, except for UAR, which had to be retrieved from the bottle with a needle and syringe, and UL in EtOH, onto which the grids were laid with the sample‐bearing side upwards since they sank under the stain droplet instead of floating. The grids were then incubated with the stain solution for 1 min at room temperature and subsequently blotted perpendicularly on Whatman paper. Then the grids were laid twice with sections pointing downward onto a ≈300–500 µL MilliQ water droplet for 30 s to wash away stain precipitates, blotted as above, and air‐dried. Several grids were prepared per stain, and representative images are shown in the Results section. In summary, the stains were compared using the same epoxy block for the sections and a consistent staining protocol, including equal positive‐staining time. TEM micrographs were acquired for particle samples with a Zeiss EM 900 standard TEM (Carl Zeiss Microscopy GmbH, Germany) with constant hardware and software settings as described in detail above.

### Synthetic Image Generation and Frequency Domain Analysis

Synthetic images of ferritin rings were generated using a custom Python module, available at doi.org/10.5281/zenodo.14944676 (v0.1). Ring centers were first placed using a naïve dart throwing algorithm^[^ ^105^ ^]^ with a rejection distance equal to the ring outer radius. The number of rings and average intermolecular distance were controlled by imposing the areal fraction covered by the rings and the core). To mimic the experimental acquisition conditions, the generated images were then passed through a low‐pass Gaussian filter in the frequency domain, and Gaussian white noise was added to the filtered image. An overview of the synthetic data generation is shown in Figures S1 and S2 (Supporting Information). Final parameters used for the synthetic data generation were: areal fraction of 0.35, filter standard deviation of 1.5, noise standard deviation of 5. Ferritin ring size and grayscale values were set using the UA images as a reference (outer diameter: 11.0 nm,^[^ ^75^ ^]^ inner diameter: 5.4 nm from the images).

The power spectrum of both synthetic and experimental images was calculated with the fast Fourier transform in the SciPy implementation.^[^ ^106^ ^]^ The averaged power spectrum was then calculated as the radial average across the power spectra of several images of the same stain and magnification (from a minimum n = 1 for Lc to a maximum of n = 10 for synthetic data). The code used for this operation is also available at doi.org/10.5281/zenodo.14944676 (v0.1).

### Statistical Analysis and Programs

The TEM images and data of size measurements were not transformed, normalized, changed in contrast/brightness/resolution, etc., and no outliers were excluded. Several grids per sample and stain were prepared and imaged (n = 3). Particle diameters were measured along the longest axis of the particles using the TEM user interface iTEM 5.1 (Build 1700, Olympus) and ImageJ 1.54b (NIH, USA) software, plotted and statistically analyzed using GraphPad Prism 10.3.1 (509) software. The size measurement plots show mean ± SD and n = 18–28 particles were measured per stain for the outer ferritin diameter. For PMMA outer diameter measurements, n = 9‐28 particles were measured per stain (lower number of particles due to lower visibility of particle borders with some stains). Grammarly and ChatGPT 4.0 were used for language improvement (spelling, grammar checks). GitHub Copilot was used for code completion. The Table of Contents graphic, Figure 1, the recurring sample icons from Table 2, and the cell scheme in Figures 11 and 12 were created using BioRender with the licenses of the first author Vera Kissling (https://BioRender.com/m04y741, https://BioRender.com/o59o885, https://BioRender.com/b14e135, https://BioRender.com/x75e711).

## Conflict of Interest

The authors declare no conflict of interest.

## Author Contributions

V.M.K. performed conceptualization, project administration, methodology, investigation, supervision of S.E., validation, formal analysis, data curation, visualization, writing ‒ original draft preparation, and writing ‒ review and editing. S.E. provided methodology, investigation and validation under the supervision of V.M.K. and P.W. for psTEM of cells, as well as writing ‒ review and editing. D.B. contributed with methodology, formal analysis, validation, software, resources, data curation and visualization of the synthetic image generation and frequency domain analysis, as well as writing ‒ review and editing. G.C. provided methodology, investigation, validation, supervision, funding acquisition and resources for the amyloid fibril work in this study, as well as writing ‒ review and editing. P.W. provided supervision of V.M.K. and S.E., funding acquisition, resources, and writing ‒ review and editing.

## Supporting information



Supporting Information

## Data Availability

All data are available from the corresponding authors upon reasonable request. The original code reported in this paper is available at doi.org/10.5281/zenodo.14944676 (v0.1).
