# Supplementary material for: Systematic Comparison of Commercial Uranyl‐Alternative Stains for Negative‐ and Positive‐Staining Transmission Electron Microscopy of Organic Specimens
Source: Adv Healthc Mater. 2025 Apr 29;14(16):2404870. doi: 10.1002/adhm.202404870 (PMC12184085; doi:10.1002/adhm.202404870)
Supplement: Supplementary file 1 — Supporting Information [file ADHM-14-0-s001.docx]

Supporting Information

**Systematic Comparison of Commercial Uranyl-Alternative Stains for Negative- and Positive-Staining Transmission Electron Microscopy of Organic Specimens**

***Vera M. Kissling^*^, Stephanie Eitner, Davide Bottone, Gea Cereghetti, Peter Wick^*^***

^#^ Co-corresponding authors

***Figure S1.*** *Comparison of synthetic images of ferritin rings (****a,c,e,g****) and their respective power spectra (****b,d,f,h****)* *generated with different parameters to simulate experimental nsTEM images as used in Figure 9.* ***a-h:*** *Ring centers were placed randomly in a blank image to create a synthetic grey-scale image with light ferritin-like rings on a darker background as in experimental nsTEM images* ***(a,c,e,g)****. A lower (0.10;* ***a,e****) and a higher (0.35;* ***c,g****) areal fraction parameter was applied to the images to assess the effect of the number of ferritin rings and their intermolecular distances (see Figure S2) in the image on the power spectrum, since in experimental nsTEM images (Figure 9) the density of ferritin rings in the image might slightly vary due to uncontrollable particle distribution differences on the grid. In* ***e-h****, the same images and their power spectra are shown after applying a Gaussian smoothing low pass filter (filter_sigma=1.5 in the generating algorithm) to remove the high frequency components of the synthetic image (outer rings in the power spectrum visible in unfiltered panels* ***b,d****) that are not present in the experimental data due to the microscope characteristics (compare* ***f,h*** *of the filtered synthetic data with the power spectra of experimental data in Figure 9). Moreover, a zero-mean Gaussian additive noise filter (noise_sigma=5 in the generating algorithm) was applied to introduce noise in the filtered synthetic images, which is normally present in experimental nsTEM data (see graininess in filtered synthetic images with noise in* ***e,g*** *compared to unfiltered synthetic images without noise in* ***a,c****).* ***i-j:*** *Comparison of the radially averaged power spectrum of the unfiltered synthetic images without noise* ***(i)*** *and the filtered synthetic images with noise* ***(j)*** *at different areal fractions. Notably, increasing the areal fraction (i.e. increasing the number of ferritin rings and lowering their intermolecular distance) has a negligible effect on the image power spectrum (only changing the intensity of the spectrum peaks, but not their frequency i.e. peak positions; compare also panels* ***b*** *and* ***d*** *or* ***f*** *and* ***h****). This suggests that slight variations in the ferritin density in the experimental nsTEM images and synthetic data do not significantly influence the peak positions of their respective power spectra. Moreover,* ***i*** *and* ***j*** *show that filtering and noise introduction attenuated the high frequency components in the power spectra, closely simulating the power spectra of the experimental nsTEM images in Figure 9.*


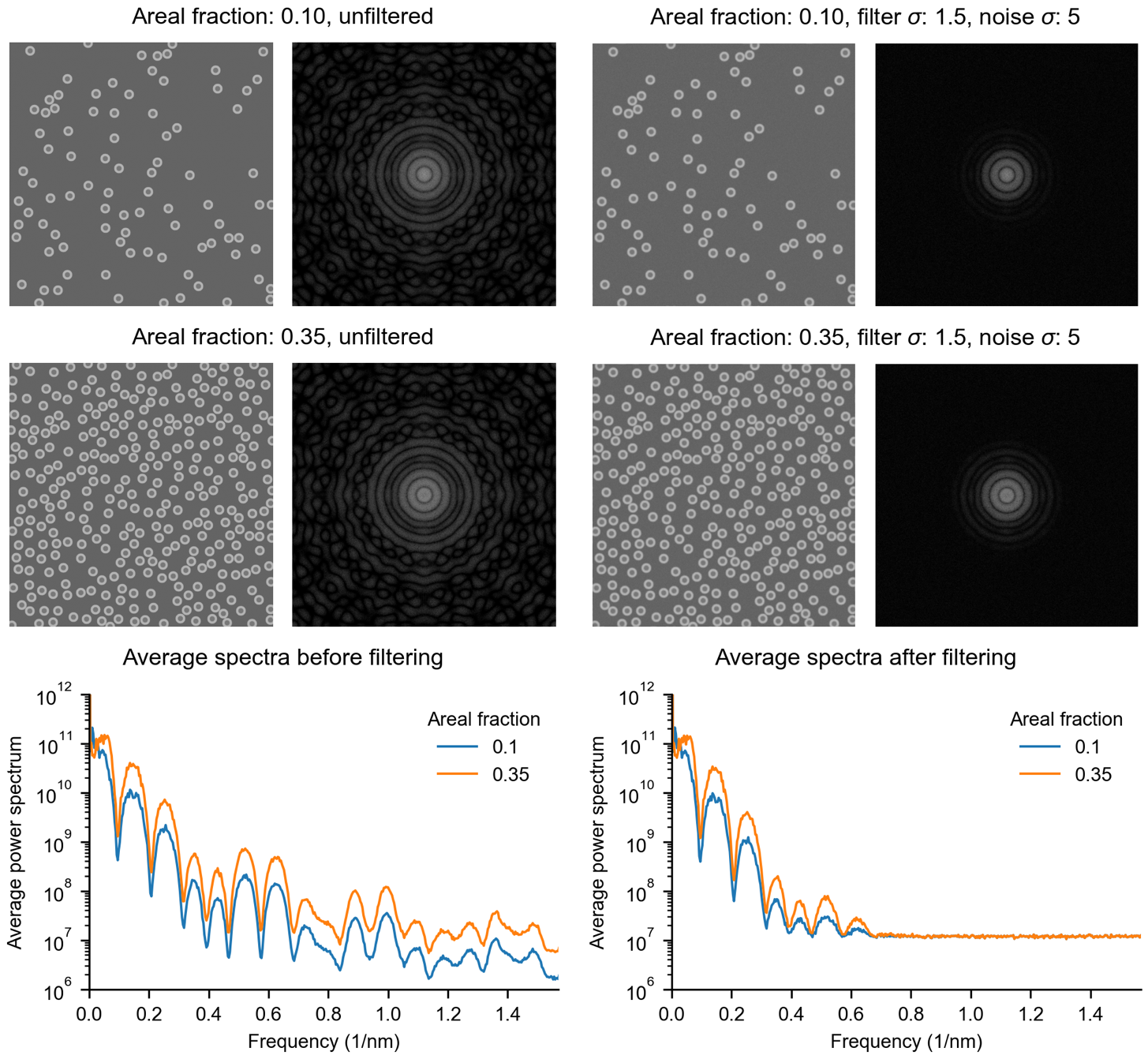


**a**

**c**

**e**

**b**

**d**

**f**

**g**

**h**

**i**

**j**

**
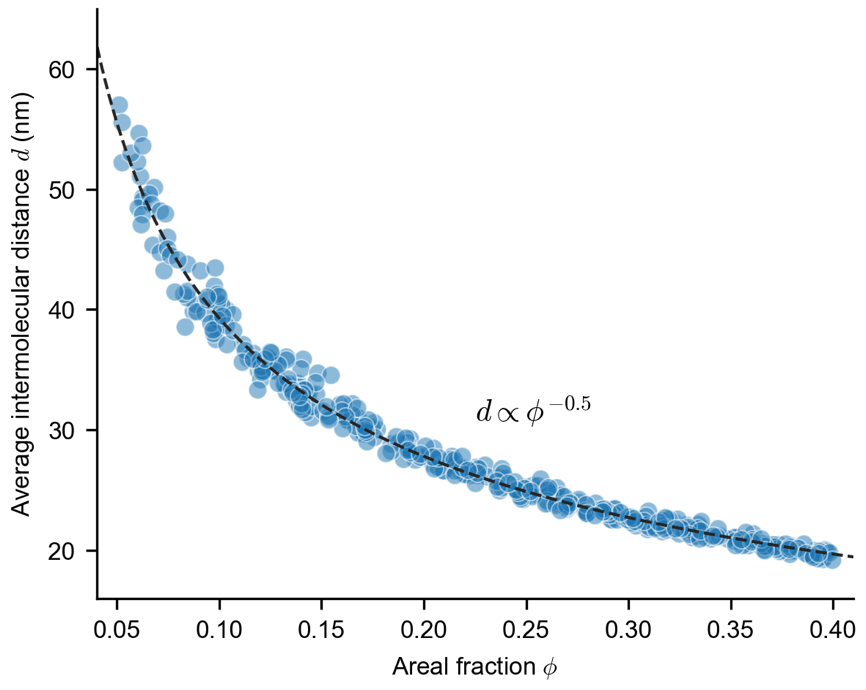
**

***Figure S2.*** *Average intermolecular distance of simulated ferritin rings as a function of areal fraction* $\phi$*, as a result of 400 runs of the generative algorithm (min_ring_spacing=0). The average intermolecular distance is defined as the average distance between the center of a ring and its nearest neighbors, identified as the opposite vertices of its Delauney triangles. The areal fraction reported is the actual areal fraction of ferritin in the image, which may be slightly lower than the nominal one used for generating the image due to rings that are not placed fully inside the image. The average intermolecular distance is proportional to* $\phi^{-0.5}$*, therefore it can effectively be controlled by setting the areal fraction.*
